# Supplementary material for: Diagnostic implications of pitfalls in causal variant identification based on 4577 molecularly characterized families
Source: Nat Commun. 2023 Aug 29;14:5269. doi: 10.1038/s41467-023-40909-3 (PMC10465531; doi:10.1038/s41467-023-40909-3)
Supplement: Supplementary file 1 — Supplementary Information [file 41467_2023_40909_MOESM1_ESM.pdf]

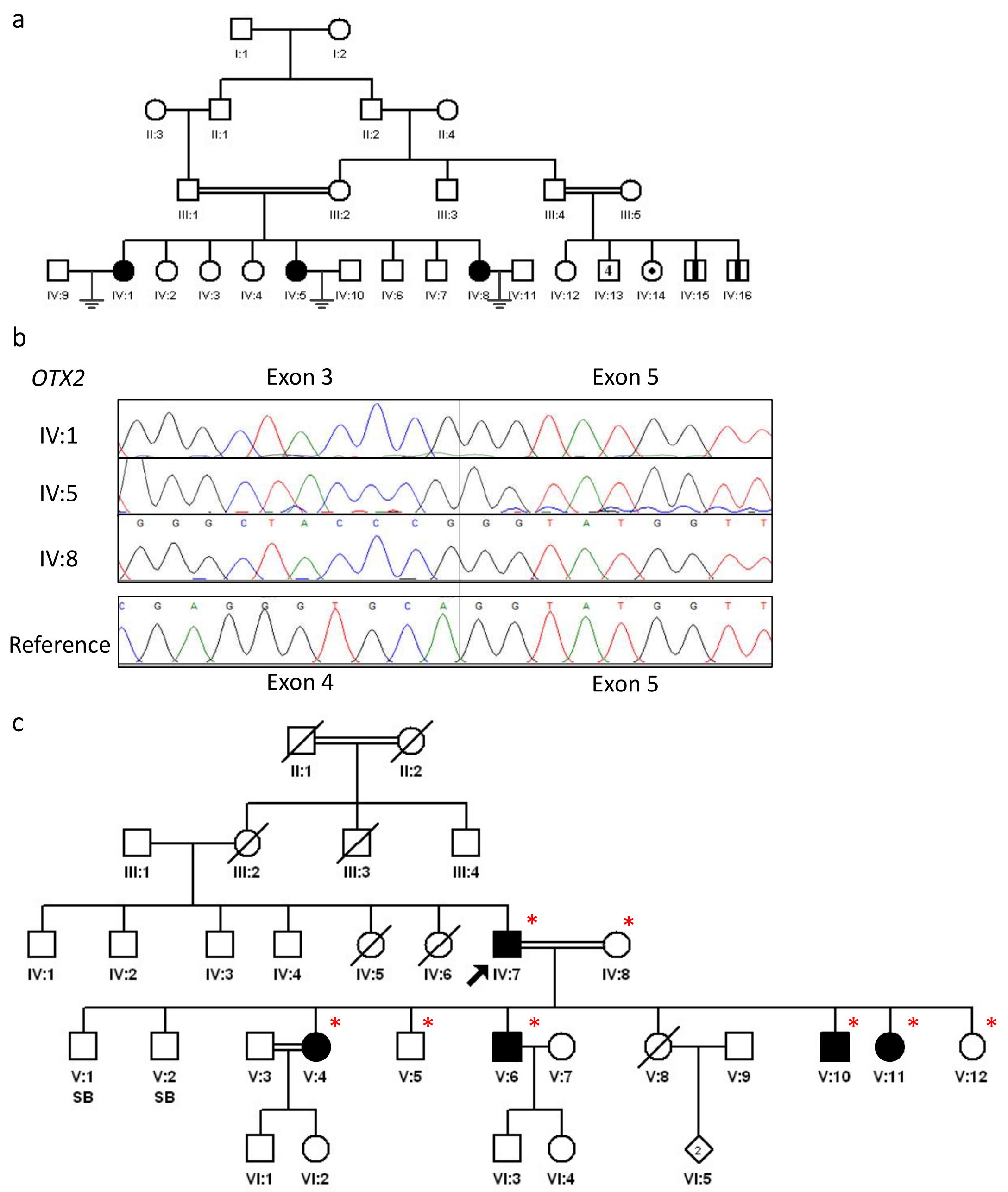

Supplementary Figure 1: Examples of families with novel allelic disorders and highly variable phenotypes.

a) Pedigree of F141 with three sisters (IV:1, IV:5, and IV9) affected with retinal dystrophy and infertility. Their maternal cousins have two brothers (IV:15 and IV:16) affected with Usher syndrome and a sister with epilepsy (IV:12). b) RT-PCR experiment using RNA extracted from the three affected sisters showed aberrant transcript in *OTX2* compared to control. Sequencing revealed that in the three affected sisters, there is skipping of exon 4 in *OTX2* compared to control. c) Pedigree of F7902 with a variant in *ALDOB*:NM_000035.4:c.448G>C;p.(Ala150Pro) displaying a surprisingly mild form of fructose intolerance (* indicates individuals with available DNA for testing).

a

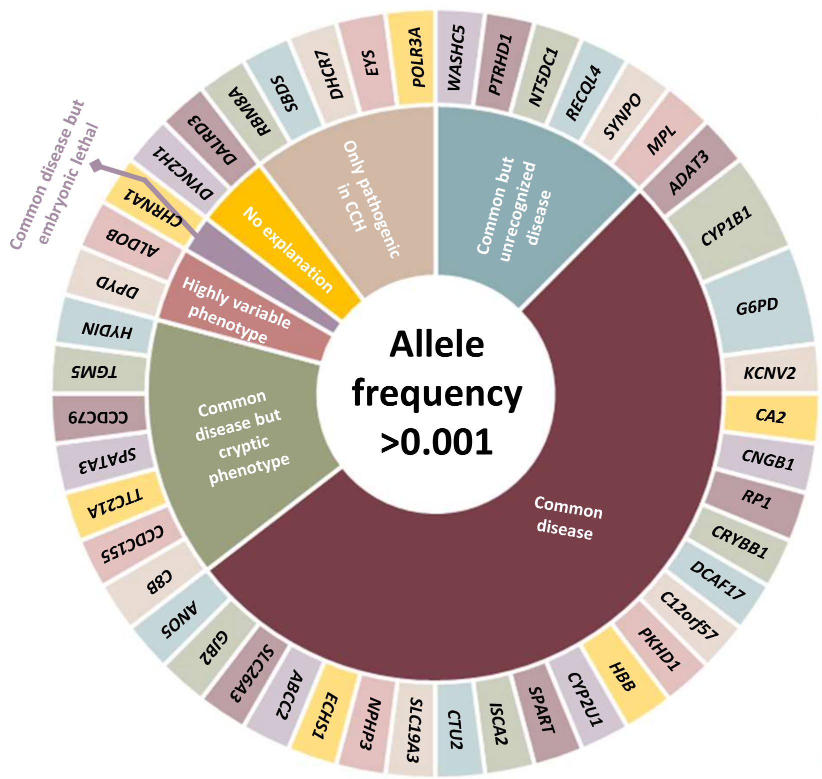

b

Justification	Allele frequency	Gene	Variant	# of families	Family IDs
Common but unrecognized disease	0.002435	*PTRHD1*	NM_001013663.1:c.365G>A;p.(Arg122Gln)	2	F1060, F5150
	0.001623	*RECQL4*	NM_004260.2:c.3128T>A;p.(Leu1043*)	1	F8226
	0.001127	*WASHC5*	NM_014846.3:c.2849A>G;p.(Lys950Arg)	1	F408
	0.001037	*SYNPO*	NM_001166209.1:c.2540C>T;p.(Pro847Leu)	1	F5878
	0.018073	*MPL*	NM_005373.3:c.317C>T;p.(Pro106Leu)	1	F9145
	0.001127	*NT5DC1*	NM_152729.3:c.1114T>C;p.(Ser372Pro)	1	F6301
Common disease	0.017931	*HBB*	NM_000518.4:c.20A>T;p.(Glu7Val)	3	F527, F4367, F4691
	0.012548	*G6PD*	NM_000402.4:c.653C>T;p.(Ser218Phe)	4	F5495, F8565, F6291, F8214
	0.012146	*CYP1B1*	NM_000104.3:c.182G>A;p.(Gly61Glu)	82	F76, F193, F205, F273, F368, F373, F375, F437, F438, F449, F457, F503, F504, F505, F514, F516, F517, F552, F553, F554, F562, F565, F566, F567, F569,F570, F579, F580, F597, F601, F608, F609, F643, F647,F649, F705, F785, F792, F806, F812, F843, F884, F923, F981, F1029, F1040, F4982, F1056, F1069, F1121, F1122, F1123, F1220, F1222,F1225, F1244, F1357, F1371, F1416, F1434, F1744, F1748, F1754, F1774, F1795, F1805, F1834, F1870, F1890, F1891, F2082, F2249, F2298, F2486, F2570, F2578, F2725, F3200, F4392, F4750, F4838, F8304
	0.009972	*G6PD*	NM_000402.3:c.233T>C; p.(Ile78Thr)	12	F900, F1793, F3182, F3310, F3432, F3612, F3908, F3924, F4138, F4424, F5028, F6352
	0.006765	*CNGB1*	NM_001297.4:c.2957A>T;p.(Asn986Ile)	4	F55, F2577, F2674, F2943
	0.005097	*ANO5*	NM_213599.2:c.172C>T;p.(Arg58Trp)	1	F6401
	0.002944	*ADAT3*	NM_138422.2:c.430G>A;p.(Val144Met)	19	F4, F72, F387, F619, F771, F1321, F2053, F2247, F2282, F2752, F3052, F3190, F3592, F4845, F5467, F8487, F6307, F6337, F6422
	0.002618	*CA2*	NM_000067.2:c.232+1G>A	9	F37, F1338, F2563, F2790, F3964, F4904, F5381, F5453, F6429
	0.002618	*SLC26A3*	NM_000111.2:c.559G>T;p.(Gly187*)	2	F6300, F7894
	0.002616	*PKHD1*	NM_138694.3:c.4870C>T;p.(Arg1624Trp)	6	F3304, F3680, F6585, F6732, F7541, F7542
	0.0023	*CYP2U1*	NM_183075.2:c.947A>T;p.(Asp316Val)	3	F590, F1104, F4124
	0.002074	*ABCC2*	NM_000392.3:c.2273G>T;p.(Gly758Val)	2	F5988, F6359
	0.001849	*RP1*	NM_006269.1:c.606C>A;p.(Asp202Glu)	4	F121, F537, F5841, F4277
	0.001713	*GJB2*	NM_004004.6:c.35delG;p.(Gly12Valfs*2)	1	F6386
	0.001489	*C12orf57*	NM_138425.4:c.1A>G;p.?	9	F342, F1086, F1418, F1841, F4311, F4334, F5516, F5954, F6281
	0.001488	*CRYBB1*	NM_001887.3:c.171delG;p.(Asn58Thrfs*107)	17	F23, F950, F1175, F1372, F1417, F1716, F1749, F1761, F1766, F1833, F1861, F3255, F3374, F4864, F4945, F5135, F5136
	0.001353	*SLC19A3*	NM_025243.3:c.1264A>G;p.(Thr422Ala)	4	F2830, F5413, F6400, F7923
	0.001263	*KCNV2*	NM_133497.3:c.427G>T;p.(Glu143*)	14	F185, F260, F543, F671, F702, F869, F915, F1229, F1348, F2327, F2694, F3356, F3392, F6460
	0.001263	*DCAF17*	NM_025000.4:c.436delC;p.(Ala147Hisfs*9)	7	F953, F1319, F2270, F2959, F3412, F3862, F8505
	0.001263	*NPHP3*	NM_153240.4:c.2694-2_2694-1delAG	10	F3637, F4198, F5994, F6592, F6676, F6928, F7524, F7531, F7532, F7533
	0.001127	*CYP1B1*	NM_000104.3:c.1405C>T;p.(Arg469Trp)	7	F449, F504, F566, F1122, F1222, F1779, F4838
	0.001127	*ECHS1*	NM_004092.4:c.88+5G>A	1	F5351
	0.001082	*SPART*	NM_001142294.1:c.988A>G;p.(Met330Val)	4	F1095, F4944, F5317, F5887
	0.001082	*CTU2*	NM_001012762.1:c.873G>A;r.738_873del;p.(Thr247Alafs*21)	5	F2713, F4238, F4491, F5528, F7794
	0.001039	*ISCA2*	NM_194279.4:c.229G>A;p.(Gly77Ser)	3	F1794, F3950, F8747
Common disease but cryptic phenotype	0.001983	*HYDIN*	NM_001270974.1:c.15217A>G;p.(Lys5073Glu)	1	F7426
	0.001533	*TTC21A*	NM_145755.2:c.1997A>G;p.(Asn666Ser)	1	F7166
	0.001443	*SPATA3*	NM_139073.3:c.373C>A;p.(Arg125Ser)	1	F7276
	0.001398	*CCDC155*	NM_144688.5:c.302T>C;p.(Met101Thr)	1	F6517
	0.001127	*CCDC79*	NM_001136505.1:c.733G>A;p.(Gly245Arg)	2	F7337, F7361
	0.010242	*TGM5*	NM_201631.4:c.1335G>C;p.(Lys445Asn)	1	F9185
	0.002615	*C8B*	NM_000066.2:c.1282C>T;p.(Arg428*)	1	F8448
Common disease but embryonic lethal	0.001262	*CHRNA1*	NM_000079.3:c.254T>C;p.(Leu85Pro)	2	F8595, F5922
Highly variable phenotype	0.003066	*DPYD*	NM_000110.3:c.257C>T;p.(Pro86Leu)	2	F169, F3966
	0.002705	*ALDOB*	NM_000035.3:c.448G>C;p.(Ala150Pro)	1	F7902
No explanation	0.001397	*DYNC2H1*	NM_001080463.1:c.1151C>T;p.(Ala384Val)	1	F5904
	0.001082	*DALRD3*	NM_001276405.1:c.1251C>A;p.(Tyr417*)	1	F6574
Only pathogenic in CCH	0.014411	*RBM8A*	NM_005105.4:c.*6C>G	1	F5228
	0.003652	*DHCR7*	NM_001163817.2:c.1A>G;p.?	1	F6277
	0.001082	*SBDS*	NM_016038.2:c.258+2T>C	1	F4212
	0.007348	*EYS*	NM_001142800.2:c.2137+1G>A	2	F8348 and F2084
	0.005418	*POLR3A*	NM_007055.4:c.1909+22G>A	1	F274

Supplementary Figure 2: Classification of disease-causing variants with Allele frequency above cut-off identified in our cohort.

a) Donut plot showing the contribution of each class of variants with AF above cut-off and the genes within these classes. b) Table detailing the local frequency of each variant, number of families identified with this variant, and the family IDs.

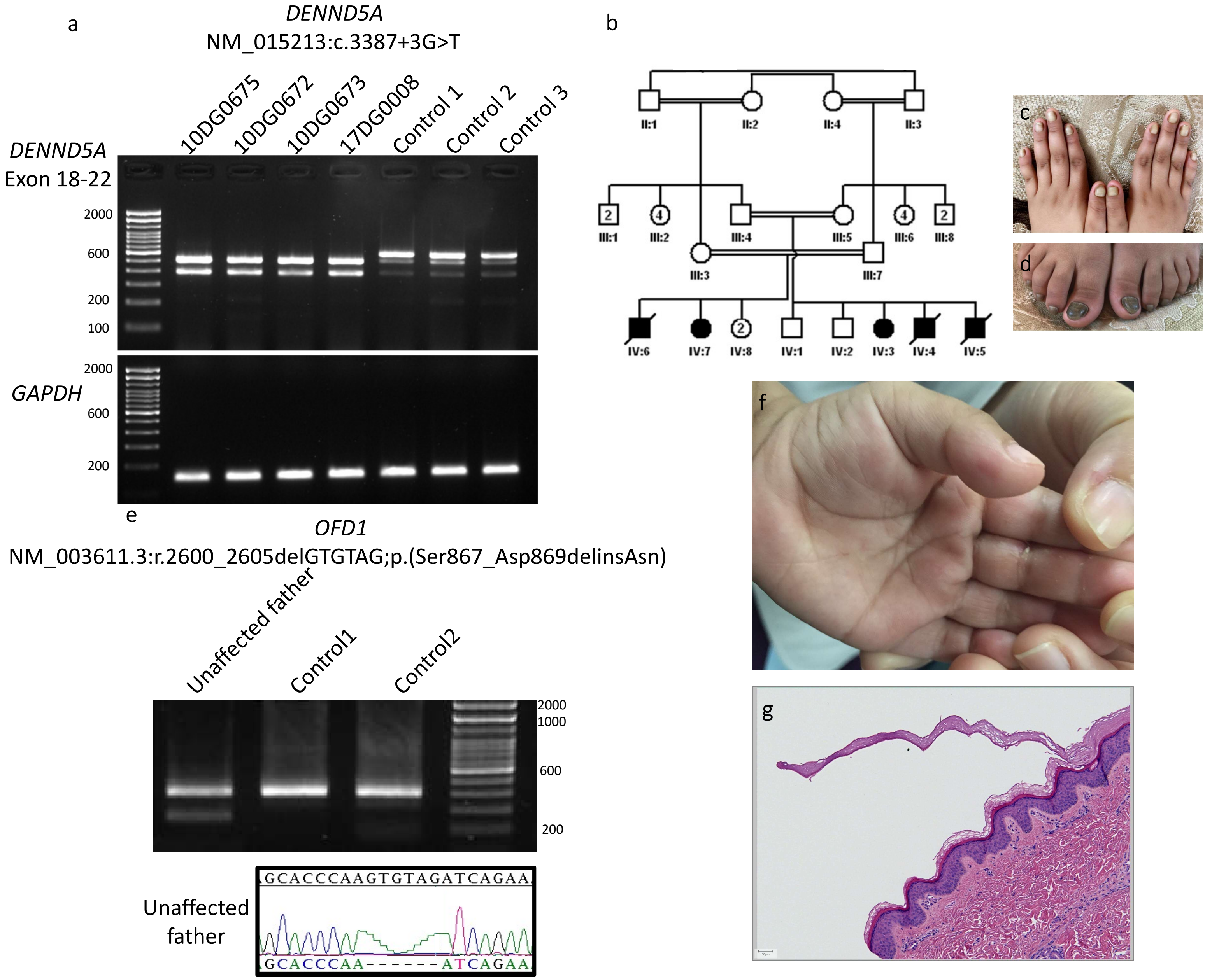

Supplementary Figure 3: Examples of families with variant-related challenges.

a) RT-PCR gel image showing aberrant transcript in individuals with a homozygous *DENND5A* splice variant compared to controls. The same aberrant transcript is observed in the unaffected sister 10DG0675. b) pedigree of family F5409 showing a deceiving penetrance situation where some affected individuals (IV:3 and IV:7) were diagnosed with club nails (c and d) and others were NIHF (IV:4, IV:5, and IV6). e) RT-PCR experiment using an unaffected individual with hemizygous splicing variant in *OFD1*. Compared to control, there is an aberrant transcript that was shown to cause a small in-frame deletion. f and g) Clinical images of patient F9185 showing the skin phenotype and typical intra-epidermal cleft on histopathology, respectively. All data presented in a, e, and g are representative images of at least two experiments.

a

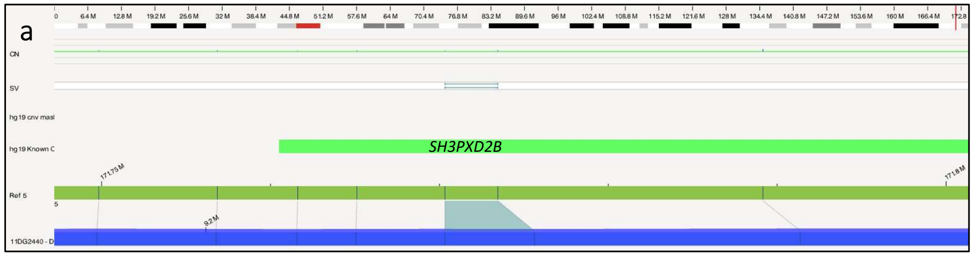

11DG2440

Type of SV	Insertion
Location	chr5:171,770,324 - 171,773,463
Size	2,157 bp
Zygosity	Homozygous
Overlapping Gene(s)	*SH3PXD2B*

b

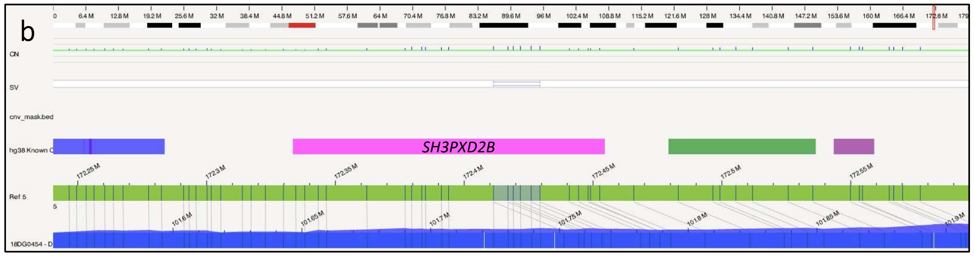

18DG0454

Type of SV	Duplication
Location	chr5:172,411,368 - 172,429,362
Size	17,994 bp
Zygosity	Homozygous
Overlapping Gene(s)	*SH3PXD2B*

c

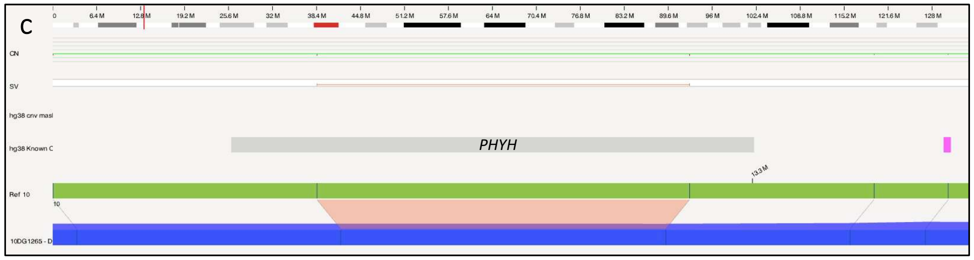

10DG1265

Type of SV	Deletion
Location	chr10:13,281,451 - 13,297,324
Size	2,019 bp
Zygosity	Homozygous
Overlapping Gene(s)	*PHYH*

d 11DG1685

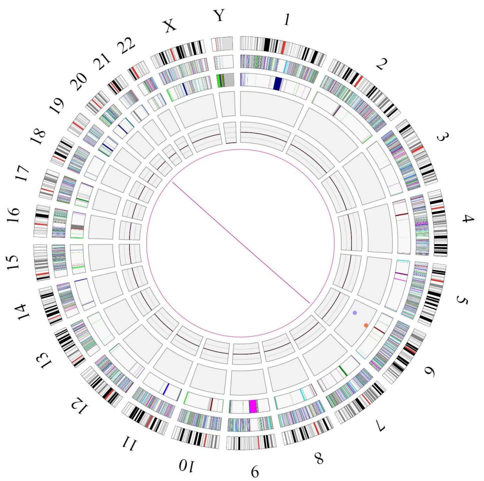

e 22DG0746

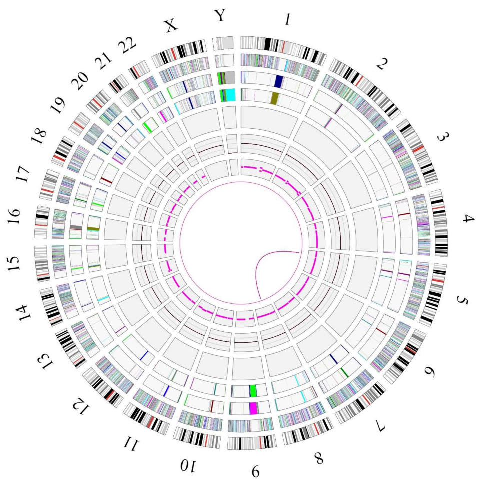

Supplementary Figure 4: Structural variants identified by optical genome mapping.

a) Family F2493 with ~2kb insertion disrupting *SH3PXD2B*. b) Family F6158 with a large duplication (~18kb) disrupting *SH3PXD2B*. c) Family F1221 with ~2kb deletion in *PHYH*. d) Family F2242 with interchromosomal translocation [GRCh37] t(6:20)(q25.1;p13) disrupting *TAB2*. e) Family F9257 solved with interchromosomal translocation [GRCh38] t(5;8)(p13.2;q13.3) disrupting *EYA1*.

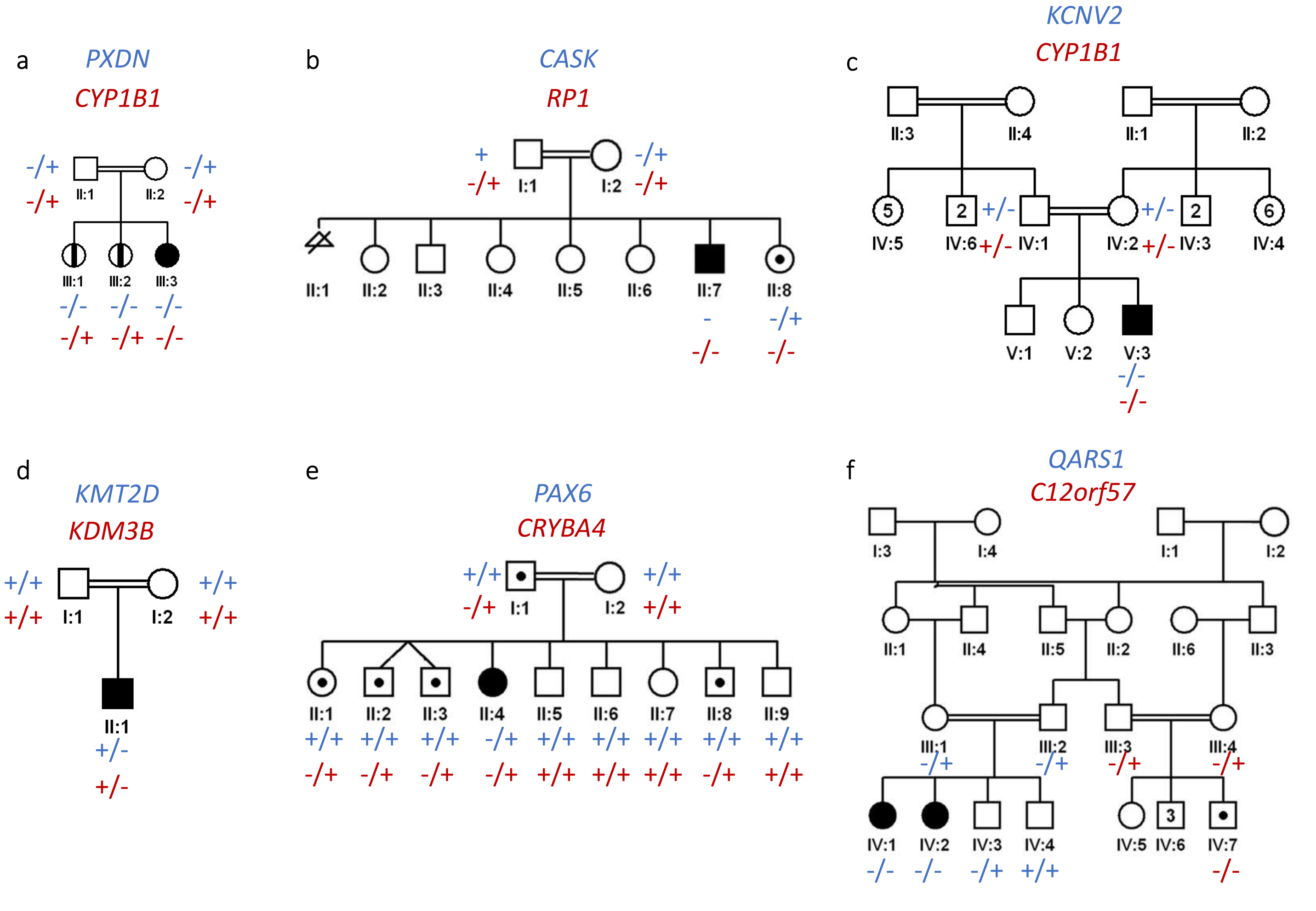

Supplementary Figure 5: Example of pedigrees showing genetic heterogeneity.

a) Pedigree of family F656 comprising three affected sisters with a homozygous variant in *PXDN* and one sister who is also homozygous for a pathogenic variant in *CYP1B1*. b) Pedigree of family F4277 where the affected index is hemizygous for *CASK*:NM_001367721.1:c.1116C>G;p.(His372Gln) and homozygous for *RP1*:NM_006269.2:c.606C>A;p.(Asp202Glu) while his sister (II:8) is homozygous for the *RP1* variant. c) Pedigree for family F3200 where individual (V:3) has homozygous pathogenic variants in *KCNV2* and *CYP1B1*. d) Pedigree of family F6353 where one affected individual has two de novo variants: *KMT2D*:NM_003482.4:c.1661delT;p.(Leu554Cysfs*376) and *KDM3B*:NM_016604.4:c.5056G>A;p.(Gly1686Arg). e) Pedigree of F4771 where one (II:4) affected has a de novo LOF variant in *PAX6* and she is also heterozygous (together with individuals I:1, II:1, II:2, II:3, and II:8) for a pathogenic variant in *CRYBA4*. f) Pedigree of family F5516 where two sisters (IV:1 and IV:2) are homozygous for *QARS1*:NM_005051.3:c.1058G>T;p.(Gly353Val) while their paternal cousin (IV:7) is homozygote for LOF variant in *C12orf57*:NM_138425.4:c.1A>G;p.?. («+» denotes WT and «-» denotes mutant allele).

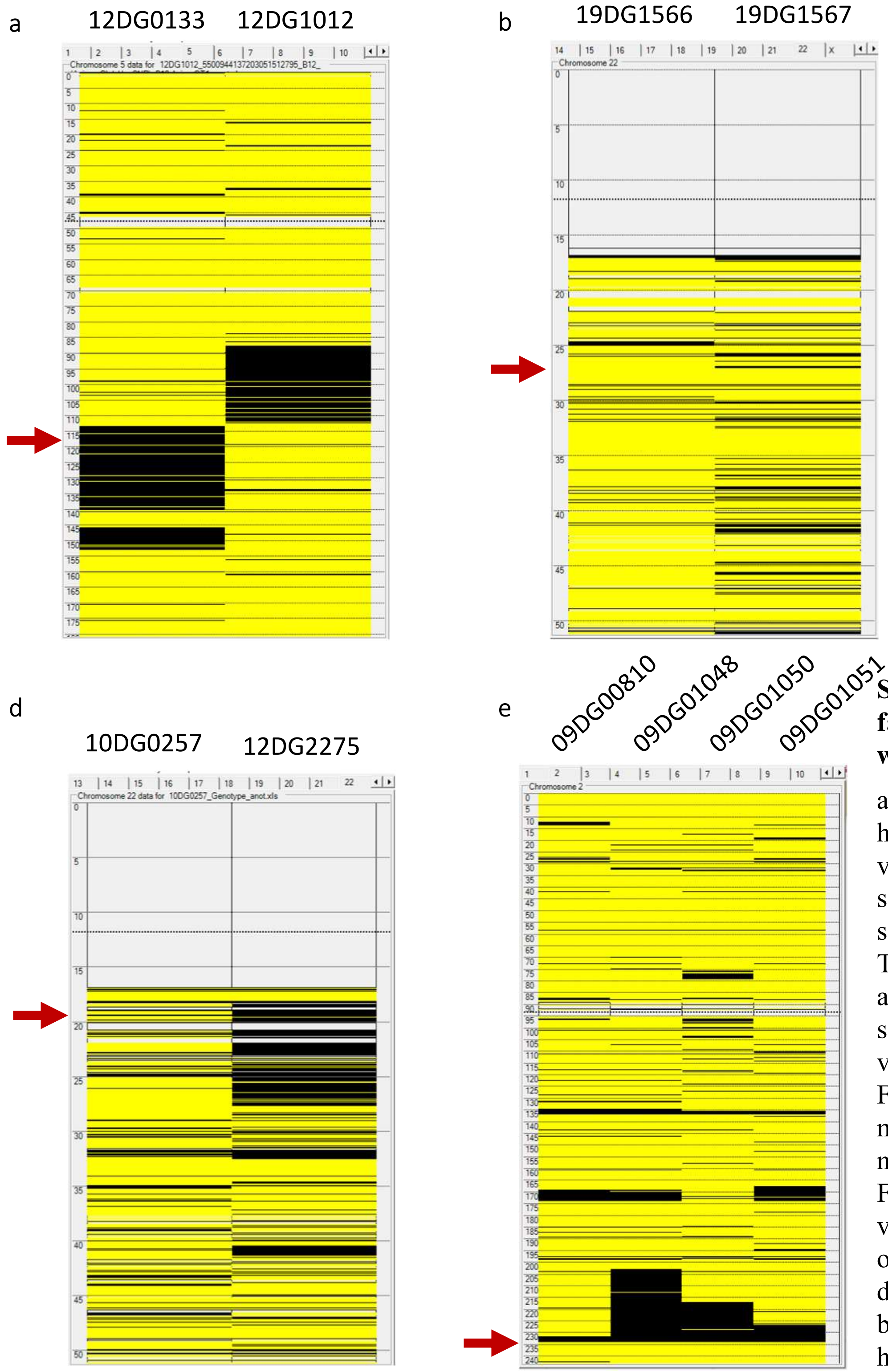

Supplementary Figure 6: Examples of families where the causal variant was not within an ROH in genotyping data.

a) Family F2573 where two affected brothers have a homozygous variant in *HSD17B4*. The variant is not within ROH in one affected sibling. b) Family F6982 where two affected siblings have a truncating variant in *CRYBB1*. The variant is not within ROH in either affected. c)Family F4177 where two affected siblings have a LOF variant in *WDR45B*. The variant is not within ROH in one affected. d) Family F849 where two brothers have a missense variant in *ATP6V1E1*. The variant is not within ROH in one affected. e) Family F473 where four siblings share a truncating variant in *PRSS56*. ROH overlap was slightly off leading to initial dismissal of the gene during mapping. Each row represents a SNP; black is homozygous and yellow is heterozygous. Red arrows denote variant location for each family.

Supplementary Methods

Ion Torrent

Select cases underwent Ion Torrent analysis using one or more of the previously designed Gene panels as in [293]. Briefly, DNA samples amplified using gene panel Primer Pools, AmpliSeq HiFi mix (Thermo Fisher, Carlsbad, CA, USA). PCR pools were combined and subjected to primer digestion with FuPa reagent (Thermo Fisher, Carlsbad, CA, USA). Pooled amplicons were ligated with universal adapters and then purified. Libraries were quantified by quantitative PCR. Normalized libraries were barcoded and pooled in equal ratios for emulsion PCR (ePCR) on an Ion OneTouch System. Then, templated Ion Sphere particles were enriched using the Ion OneTouch ES enrichment system as per manufacturer's instructions (Thermo Fisher, Carlsbad, CA, USA). The Ion PI Ion Sphere particles were processed for sequencing on the Ion Proton instrument (Thermo Fisher, Carlsbad, CA, USA).

Whole Exome Sequencing (WES)

Whole exome sequencing was performed as described previously [123]. The exome target regions were captured using platform-specific capture kits according to the recommended manufacturer's protocol. Libraries were made and were enriched for the desired target using the Illumina Exome Enrichment protocol. The final libraries were then sequenced their respective platforms to an average read depth of 81.8X. Reads were mapped against UCSC hg19 by BWA. GATK package was used for calling SNVs and Indels.

Molecular Karyotyping

The majority of simplex cases underwent Molecular Karyotyping as described [271]. Briefly, genome-wide SNP CytoScan HD array was used to perform microarray analysis to assess for genomic gains or losses. This array platform contains 2.6 million markers for CNV detection (Affymetrix). Chromosome Analysis Suite version Cyto 2.0.0.195(r5758) was used for analysis based on UCSC hg19.

Optical Genome Mapping

High molecular weight (HMW) DNA was extracted from 1.5 million patient-derived lymphoblastoid cell lines (LCLs) using the Bionano Prep SP Blood and Cell DNA Isolation Kit (Bionano Genomics, CA, USA, #80030), according to the manufacturer's recommendations. DNA quantification was performed using the Qubit dsDNA BR assay kit with a Qubit 2.0 Fluorometer (Thermo Fisher Scientific). A total of 750ng of HMW DNA was labelled using the Bionano Prep Direct Label and Stain DLS DNA Kit (Bionano Genomics, #80005), following the manufacturer's protocol. The HMW-labelled DNA was loaded onto the Saphyr Chip (Bionano Genomics, #20319) flow cell at a concentration of 12 ng/µl and the samples were run on Bionano Saphyr instrument, according to the manufacturer's instructions, targeting 300× human genome coverage by collecting 500 GB of data per sample [294]. All data were analyzed using Bionano Access software (v1.5) featuring both de novo variant bioinformatics pipelines (hg19 and hg38), according to the manufacturer's recommendations.

Supplementary Table 1

Primers used in this study:

Target	Forward primer	Tm	Reverse Primer	Tm	Size	Type
ABL1	CCTGAATGAAGATGAGCGCC	59.1	TCAAACTGTCTTCCCGTGGA	58.9	495	PCR
OTX2	CTGAACCTGTCCACCCCG	59.7	TGGCCACTTGTTCCACTCTC	59.9	500	RT-PCR
DKK1	CCTTGGATGGGTATTCCAGA	59.7	CCTGAGGCACAGTCTGATGA	60.0	221	qRT-PCR
DENND5A	TGGCTGGTGGAGTATGTGAT	59.0	TGAAACTTGCCATCCTTGCC	63.4	578	RT-PCR
OFD1	AATCTGCAGGGAACATGC	59	TCTCTAAAGGATTTTCAC	59	343	RT-PCR
GAPDH	GGTGAAGGTCGGAGTCAAC	59	ATGGGTGGAATCATATTGGA	58	140	RT-PCR and qRT-PCR

Supplementary References

1. Patel N, *et al.* Expanding the clinical, allelic, and locus heterogeneity of retinal dystrophies. *Genet Med* **18**, 554-562 (2016).
2. Shaheen R, *et al.* Accelerating matchmaking of novel dysmorphology syndromes through clinical and genomic characterization of a large cohort. *Genet Med* **18**, 686-695 (2016).
3. Anazi S, *et al.* Clinical genomics expands the morbid genome of intellectual disability and offers a high diagnostic yield. *Mol Psychiatry* **22**, 615-624 (2017).
4. Alsaif HS, *et al.* Congenital glaucoma and CYP1B1: an old story revisited. *Hum Genet* **138**, 1043-1049 (2019).
5. Al-Dosari MS, Shaheen R, Colak D, Alkuraya FS. Novel CENPJ mutation causes Seckel syndrome. *J Med Genet* **47**, 411-414 (2010).
6. Maddirevula S, *et al.* Exploiting the Autozygome to Support Previously Published Mendelian Gene-Disease Associations: An Update. *Front Genet* **11**, 580484 (2020).
7. Alazami AM, *et al.* Accelerating novel candidate gene discovery in neurogenetic disorders via whole-exome sequencing of prescreened multiplex consanguineous families. *Cell Rep* **10**, 148-161 (2015).
8. Anazi S, *et al.* Expanding the genetic heterogeneity of intellectual disability. *Hum Genet* **136**, 1419-1429 (2017).
9. Aldahmesh MA, Khan AO, Mohamed J, Alkuraya FS. Novel recessive BFSP2 and PITX3 mutations: insights into mutational mechanisms from consanguineous populations. *Genet Med* **13**, 978-981 (2011).
10. Maddirevula S, *et al.* Analysis of transcript-deleterious variants in Mendelian disorders: implications for RNA-based diagnostics. *Genome Biol* **21**, 145 (2020).
11. Zahrani F, Aldahmesh MA, Alshammari MJ, Al-Hazzaa SA, Alkuraya FS. Mutations in c12orf57 cause a syndromic form of colobomatous microphthalmia. *Am J Hum Genet* **92**, 387-391 (2013).
12. Bohlega S, Alazami AM, Cupler E, Al-Hindi H, Ibrahim E, Alkuraya FS. A novel syndromic form of sensory-motor polyneuropathy is linked to chromosome 22q13.31-q13.33. *Clin Genet* **79**, 193-195 (2011).
13. Loucks CM, *et al.* Matching two independent cohorts validates DPH1 as a gene responsible for autosomal recessive intellectual disability with short stature, craniofacial, and ectodermal anomalies. *Hum Mutat* **36**, 1015-1019 (2015).
14. Patel N, *et al.* Genetic investigation of 93 families with microphthalmia or posterior microphthalmos. *Clin Genet* **93**, 1210-1222 (2018).
15. Khan AO, *et al.* Genetic testing results of children suspected to have Stickler syndrome type collagenopathy after ocular examination. *Mol Genet Genomic Med* **9**, e1628 (2021).
16. Aldahmesh MA, *et al.* Genomic analysis of pediatric cataract in Saudi Arabia reveals novel candidate disease genes. *Genet Med* **14**, 955-962 (2012).
17. Shaheen R, *et al.* POC1A truncation mutation causes a ciliopathy in humans characterized by primordial dwarfism. *Am J Hum Genet* **91**, 330-336 (2012).
18. Abu-Safieh L, *et al.* Autozygome-guided exome sequencing in retinal dystrophy patients reveals pathogenetic mutations and novel candidate disease genes. *Genome Res* **23**, 236-247 (2013).
19. Sowada N, *et al.* Mutations of PTPN23 in developmental and epileptic encephalopathy. *Hum Genet* **136**, 1455-1461 (2017).
20. Alsalem AB, Halees AS, Anazi S, Alshamekh S, Alkuraya FS. Autozygome sequencing expands the horizon of human knockout research and provides novel insights into human phenotypic variation. *PLoS Genet* **9**, e1004030 (2013).
21. Maddirevula S, *et al.* Autozygome and high throughput confirmation of disease genes candidacy. *Genet Med* **21**, 736-742 (2019).
22. Seidahmed MZ, *et al.* Ritscher-Schinzel (cranio-cerebello-cardiac, 3C) syndrome: report of four new cases with renal involvement. *Am J Med Genet A* **155A**, 1393-1397 (2011).
23. Khan AO, Aldahmesh MA, Mohamed JY, Alkuraya FS. Clinical and molecular analysis of children with central pulverulent cataract from the Arabian Peninsula. *Br J Ophthalmol* **96**, 650-655 (2012).
24. Alazami AM, *et al.* Expanding the clinical and genetic heterogeneity of hereditary disorders of connective tissue. *Hum Genet* **135**, 525-540 (2016).
25. Patel N, *et al.* Novel phenotypes and loci identified through clinical genomics approaches to pediatric cataract. *Hum Genet* **136**, 205-225 (2017).

26. Aldahmesh MA*, et al.* IFT27, encoding a small GTPase component of IFT particles, is mutated in a consanguineous family with Bardet-Biedl syndrome. *Hum Mol Genet* **23**, 3307-3315 (2014).

27. Shaheen R*, et al.* Mutations in FKBP10 cause both Bruck syndrome and isolated osteogenesis imperfecta in humans. *Am J Med Genet A* **155A**, 1448-1452 (2011).

28. Al-Owain M, Al-Dosari MS, Sunker A, Shuaib T, Alkuraya FS. Identification of a novel ZNF469 mutation in a large family with Ehlers-Danlos phenotype. *Gene* **511**, 447-450 (2012).

29. Unlu G*, et al.* Phenome-based approach identifies RIC1-linked Mendelian syndrome through zebrafish models, biobank associations and clinical studies. *Nat Med* **26**, 98-109 (2020).

30. Shaheen R*, et al.* Positional mapping of PRKD1, NRP1 and PRDM1 as novel candidate disease genes in truncus arteriosus. *J Med Genet* **52**, 322-329 (2015).

31. Shaheen R*, et al.* Characterizing the morbid genome of ciliopathies. *Genome Biol* **17**, 242 (2016).

32. Abu-Safieh L*, et al.* In search of triallelism in Bardet-Biedl syndrome. *Eur J Hum Genet* **20**, 420-427 (2012).

33. Shamseldin HE*, et al.* Increasing the sensitivity of clinical exome sequencing through improved filtration strategy. *Genet Med* **19**, 593-598 (2017).

34. Al-Salem A, Alshammari MJ, Hassan H, Alazami AM, Alkuraya FS. Weaver syndrome and defective cortical development: a rare association. *Am J Med Genet A* **161A**, 225-227 (2013).

35. Alshenaifi J*, et al.* The many faces of peroxisomal disorders: Lessons from a large Arab cohort. *Clin Genet* **95**, 310-319 (2019).

36. Alazami AM, Adly N, Al Dhalaan H, Alkuraya FS. A nullimorphic ERLIN2 mutation defines a complicated hereditary spastic paraplegia locus (SPG18). *Neurogenetics* **12**, 333-336 (2011).

37. Anazi S, Al-Sabban E, Alkuraya FS. Gonadal mosaicism as a rare cause of autosomal recessive inheritance. *Clin Genet* **85**, 278-281 (2014).

38. Shaheen R*, et al.* Genomic analysis of primordial dwarfism reveals novel disease genes. *Genome Res* **24**, 291-299 (2014).

39. Maddirevula S*, et al.* Expanding the phenome and variome of skeletal dysplasia. *Genet Med* **20**, 1609-1616 (2018).

40. Al-Mayouf SM*, et al.* Loss-of-function variant in DNASE1L3 causes a familial form of systemic lupus erythematosus. *Nat Genet* **43**, 1186-1188 (2011).

41. Abu Safieh L*, et al.* Clinical and molecular characterisation of Bardet-Biedl syndrome in consanguineous populations: the power of homozygosity mapping. *J Med Genet* **47**, 236-241 (2010).

42. Shaheen R*, et al.* Identification of a novel MKS locus defined by TMEM107 mutation. *Hum Mol Genet* **24**, 5211-5218 (2015).

43. Alshammari MJ, Al-Otaibi L, Alkuraya FS. Mutation in RAB33B, which encodes a regulator of retrograde Golgi transport, defines a second Dyggve--Melchior--Clausen locus. *J Med Genet* **49**, 455-461 (2012).

44. Abu-Safieh L*, et al.* Mutation of IGFBP7 causes upregulation of BRAF/MEK/ERK pathway and familial retinal arterial macroaneurysms. *Am J Hum Genet* **89**, 313-319 (2011).

45. Alkuraya H*, et al.* Phenotypic delineation of the retinal arterial macroaneurysms with supravalvular pulmonic stenosis syndrome. *Clin Genet* **97**, 447-456 (2020).

46. Bhoj EJ*, et al.* Mutations in TBCK, Encoding TBC1-Domain-Containing Kinase, Lead to a Recognizable Syndrome of Intellectual Disability and Hypotonia. *Am J Hum Genet* **98**, 782-788 (2016).

47. Abualsaud D, Hashem M, AlHashem A, Alkuraya FS. Survey of disorders of sex development in a large cohort of patients with diverse Mendelian phenotypes. *Am J Med Genet A* **185**, 2789-2800 (2021).

48. Alrakaf L*, et al.* Further delineation of Temtamy syndrome of corpus callosum and ocular abnormalities. *Am J Med Genet A* **176**, 715-721 (2018).

49. Shamseldin HE*, et al.* Lethal variants in humans: lessons learned from a large molecular autopsy cohort. *Genome Med* **13**, 161 (2021).

50. Shaheen R*, et al.* Neu-Laxova syndrome, an inborn error of serine metabolism, is caused by mutations in PHGDH. *Am J Hum Genet* **94**, 898-904 (2014).

51. Al-Owain M, Alazami AM, Alkuraya FS. An autosomal recessive syndrome of severe cognitive impairment, dysmorphic facies and skeletal abnormalities maps to the long arm of chromosome 17. *Clin Genet* **80**, 489-492 (2011).

52. Palmer EE*, et al.* Neuronal deficiency of ARV1 causes an autosomal recessive epileptic encephalopathy. *Hum Mol Genet* **25**, 3042-3054 (2016).

53. Alsaleh N*, et al.* A Biallelic Variant in FRA10AC1 Is Associated With Neurodevelopmental Disorder and Growth Retardation. *Neurol Genet* **8**, e200010 (2022).

54. Elo JM*, et al.* Mitochondrial phenylalanyl-tRNA synthetase mutations underlie fatal infantile Alpers encephalopathy. *Hum Mol Genet* **21**, 4521-4529 (2012).

55. Shamseldin HE*, et al.* Genomic analysis of mitochondrial diseases in a consanguineous population reveals novel candidate disease genes. *J Med Genet* **49**, 234-241 (2012).

56. Maddirevula S, Alsaif HS, Ibrahim N, Alkuraya FS. A de novo mutation in FMR1 in a patient with intellectual disability. *Eur J Med Genet* **63**, 103763 (2020).

57. AlAbdi L*, et al.* Residual risk for additional recessive diseases in consanguineous couples. *Genet Med* **23**, 2448-2454 (2021).

58. Ramadan W*, et al.* Confirming the recessive inheritance of SCN1B mutations in developmental epileptic encephalopathy. *Clin Genet* **92**, 327-331 (2017).

59. Patel N*, et al.* A novel mechanism for variable phenotypic expressivity in Mendelian diseases uncovered by an AU-rich element (ARE)-creating mutation. *Genome Biol* **18**, 144 (2017).

60. Shaheen R*, et al.* A founder CEP120 mutation in Jeune asphyxiating thoracic dystrophy expands the role of centriolar proteins in skeletal ciliopathies. *Hum Mol Genet* **24**, 1410-1419 (2015).

61. Shaheen R*, et al.* A novel syndrome of hypohidrosis and intellectual disability is linked to COG6 deficiency. *J Med Genet* **50**, 431-436 (2013).

62. Gai X*, et al.* Mutations in FBXL4, encoding a mitochondrial protein, cause early-onset mitochondrial encephalomyopathy. *Am J Hum Genet* **93**, 482-495 (2013).

63. Alazami AM*, et al.* Mutation in ADAT3, encoding adenosine deaminase acting on transfer RNA, causes intellectual disability and strabismus. *J Med Genet* **50**, 425-430 (2013).

64. Patel N*, et al.* Mutations in known disease genes account for the majority of autosomal recessive retinal dystrophies. *Clin Genet* **94**, 554-563 (2018).

65. Aldeeri AA, Alazami AM, Hijazi H, Alzahrani F, Alkuraya FS. Excessively redundant umbilical skin as a potential early clinical feature of Morquio syndrome and FKBP14-related Ehlers-Danlos syndrome. *Clin Genet* **86**, 469-472 (2014).

66. Braun DA*, et al.* Mutations in multiple components of the nuclear pore complex cause nephrotic syndrome. *J Clin Invest* **128**, 4313-4328 (2018).

67. Shamseldin HE, Swaid A, Alkuraya FS. Lifting the lid on unborn lethal Mendelian phenotypes through exome sequencing. *Genet Med* **15**, 307-309 (2013).

68. Bouasker S*, et al.* Bi-allelic variants in WNT7B disrupt the development of multiple organs in humans. *J Med Genet* **60**, 294-300 (2023).

69. AlAbdi L, Alshammari M, Helaby R, Khan AO, Alkuraya FS. PMEL is mutated in oculocutaneous albinism. *Hum Genet* **142**, 139-144 (2023).

70. Aldahmesh MA*, et al.* Mutations in LRPAP1 are associated with severe myopia in humans. *Am J Hum Genet* **93**, 313-320 (2013).

71. Khan AO, Aldahmesh MA, Alkuraya FS. Clinical Characterization of LRPAP1-Related Pediatric High Myopia. *Ophthalmology* **123**, 434-435 (2016).

72. Adly N, Alhashem A, Ammari A, Alkuraya FS. Ciliary genes TBC1D32/C6orf170 and SCLT1 are mutated in patients with OFD type IX. *Hum Mutat* **35**, 36-40 (2014).

73. Magliyah MS, Alsulaiman SM, Nowilaty SR, Alkuraya FS, Schatz P. Rhegmatogenous Retinal Detachment in Nonsyndromic High Myopia Associated with Recessive Mutations in LRPAP1. *Ophthalmol Retina* **4**, 77-83 (2020).

74. Shaheen R*, et al.* Study of autosomal recessive osteogenesis imperfecta in Arabia reveals a novel locus defined by TMEM38B mutation. *J Med Genet* **49**, 630-635 (2012).

75. Eyaid W*, et al.* Transaldolase deficiency: report of 12 new cases and further delineation of the phenotype. *J Inherit Metab Dis* **36**, 997-1004 (2013).

76. Alangari A*, et al.* LPS-responsive beige-like anchor (LRBA) gene mutation in a family with inflammatory bowel disease and combined immunodeficiency. *J Allergy Clin Immunol* **130**, 481-488 e482 (2012).

77. Aldahmesh MA, Mohammed JY, Al-Hazzaa S, Alkuraya FS. Homozygous null mutation in ODZ3 causes microphthalmia in humans. *Genet Med* **14**, 900-904 (2012).

78. Aldahmesh MA, Khan AO, Meyer BF, Alkuraya FS. Mutational spectrum of SLC4A11 in autosomal recessive CHED in Saudi Arabia. *Invest Ophthalmol Vis Sci* **50**, 4142-4145 (2009).

79. Shamseldin HE, Faden MA, Alashram W, Alkuraya FS. Identification of a novel DLX5 mutation in a family with autosomal recessive split hand and foot malformation. *J Med Genet* **49**, 16-20 (2012).

80. Shamseldin HE, Elfaki M, Alkuraya FS. Exome sequencing reveals a novel Fanconi group defined by XRCC2 mutation. *J Med Genet* **49**, 184-186 (2012).

81. El-Hattab AW*, et al.* Molecular and clinical spectra of FBXL4 deficiency. *Hum Mutat* **38**, 1649-1659 (2017).

82. Dressendorfer RH. Acute reduction in maximal oxygen uptake after long-distance running. *Int J Sports Med* **12**, 30-33 (1991).

83. Ghazi NG*, et al.* Treatment of retinitis pigmentosa due to MERTK mutations by ocular subretinal injection of adeno-associated virus gene vector: results of a phase I trial. *Hum Genet* **135**, 327-343 (2016).

84. Shamseldin HE*, et al.* Mutations in DDX59 implicate RNA helicase in the pathogenesis of orofaciodigital syndrome. *Am J Hum Genet* **93**, 555-560 (2013).

85. Reynolds JJ*, et al.* Mutations in DONSON disrupt replication fork stability and cause microcephalic dwarfism. *Nat Genet* **49**, 537-549 (2017).

86. El-Hattab AW*, et al.* ADAT3-related intellectual disability: Further delineation of the phenotype. *Am J Med Genet A* **170A**, 1142-1147 (2016).

87. Shamseldin HE*, et al.* The morbid genome of ciliopathies: an update. *Genet Med* **22**, 1051-1060 (2020).

88. Aldahmesh MA, Khan AO, Mohamed JY, Alghamdi MH, Alkuraya FS. Identification of a truncation mutation of acylglycerol kinase (AGK) gene in a novel autosomal recessive cataract locus. *Hum Mutat* **33**, 960-962 (2012).

89. Aldahmesh MA*, et al.* Molecular characterization of retinitis pigmentosa in Saudi Arabia. *Mol Vis* **15**, 2464-2469 (2009).

90. Pretorius PR, Aldahmesh MA, Alkuraya FS, Sheffield VC, Slusarski DC. Functional analysis of BBS3 A89V that results in non-syndromic retinal degeneration. *Hum Mol Genet* **20**, 1625-1632 (2011).

91. Al-Dosari MS*, et al.* Mutation in MPDZ causes severe congenital hydrocephalus. *J Med Genet* **50**, 54-58 (2013).

92. Awad S*, et al.* Mutation in PHC1 implicates chromatin remodeling in primary microcephaly pathogenesis. *Hum Mol Genet* **22**, 2200-2213 (2013).

93. Alazami AM*, et al.* Mutations in C2orf37, encoding a nucleolar protein, cause hypogonadism, alopecia, diabetes mellitus, mental retardation, and extrapyramidal syndrome. *Am J Hum Genet* **83**, 684-691 (2008).

94. Anazi S, Alshammari M, Moneis D, Abouelhoda M, Ibrahim N, Alkuraya FS. Confirming the candidacy of THOC6 in the etiology of intellectual disability. *Am J Med Genet A* **170A**, 1367-1369 (2016).

95. Shaheen R*, et al.* Genomic and phenotypic delineation of congenital microcephaly. *Genet Med* **21**, 545-552 (2019).

96. Shaheen R*, et al.* The genetic landscape of familial congenital hydrocephalus. *Ann Neurol* **81**, 890-897 (2017).

97. Seidahmed MZ*, et al.* A novel syndrome of lethal familial hyperekplexia associated with brain malformation. *BMC Neurol* **12**, 125 (2012).

98. Salih MA*, et al.* Mutation in GM2A Leads to a Progressive Chorea-dementia Syndrome. *Tremor Other Hyperkinet Mov (N Y)* **5**, 306 (2015).

99. Bosley TM*, et al.* The clinical spectrum of homozygous HOXA1 mutations. *Am J Med Genet A* **146A**, 1235-1240 (2008).

100. Shaheen R, Al-Salam Z, El-Hattab AW, Alkuraya FS. The syndrome dysmorphic facies, renal agenesis, ambiguous genitalia, microcephaly, polydactyly and lissencephaly (DREAM-PL): Report of two additional patients. *Am J Med Genet A* **170**, 3222-3226 (2016).

101. Shaheen R*, et al.* Biallelic variants in CTU2 cause DREAM-PL syndrome and impair thiolation of tRNA wobble U34. *Hum Mutat* **40**, 2108-2120 (2019).

102. Saudi Mendeliome G. Comprehensive gene panels provide advantages over clinical exome sequencing for Mendelian diseases. *Genome Biol* **16**, 134 (2015).

103. Aldahmesh MA, Khan AO, Hijazi H, Alkuraya FS. Homozygous truncation of SIX6 causes complex microphthalmia in humans. *Clin Genet* **84**, 198-199 (2013).

104. Shaheen R, *et al.* Bi-allelic Mutations in FAM149B1 Cause Abnormal Primary Cilium and a Range of Ciliopathy Phenotypes in Humans. *Am J Hum Genet* **104**, 731-737 (2019).

105. Abumansour IS, *et al.* ARL6IP6, a susceptibility locus for ischemic stroke, is mutated in a patient with syndromic Cutis Marmorata Telangiectatica Congenita. *Hum Genet* **134**, 815-822 (2015).

106. Shaheen R, Alshail E, Alaqeel A, Ansari S, Hindieh F, Alkuraya FS. T (brachyury) is linked to a Mendelian form of neural tube defects in humans. *Hum Genet* **134**, 1139-1141 (2015).

107. Mohamed JY, Faqeih E, Alsiddiky A, Alshammari MJ, Ibrahim NA, Alkuraya FS. Mutations in MEOX1, encoding mesenchyme homeobox 1, cause Klippel-Feil anomaly. *Am J Hum Genet* **92**, 157-161 (2013).

108. Alazami AM, Alshammari MJ, Baig M, Salih MA, Hassan HH, Alkuraya FS. NPHP4 mutation is linked to cerebello-oculo-renal syndrome and male infertility. *Clin Genet* **85**, 371-375 (2014).

109. Hijazi H, *et al.* Pellagra-like condition is xeroderma pigmentosum/Cockayne syndrome complex and niacin confers clinical benefit. *Clin Genet* **87**, 56-61 (2015).

110. Nahorski MS, *et al.* Biallelic UFM1 and UFC1 mutations expand the essential role of ufmylation in brain development. *Brain* **141**, 1934-1945 (2018).

111. Mahajan S, *et al.* Homozygous truncating variant in MAN2A2 causes a novel congenital disorder of glycosylation with neurological involvement. *J Med Genet* **60**, 627-635 (2023).

112. Alzahrani F, Al Hazzaa SA, Tayeb H, Alkuraya FS. LOXL3, encoding lysyl oxidase-like 3, is mutated in a family with autosomal recessive Stickler syndrome. *Hum Genet* **134**, 451-453 (2015).

113. Shamseldin HE, *et al.* Molecular autopsy in maternal-fetal medicine. *Genet Med* **20**, 420-427 (2018).

114. Tallila J, Salonen R, Kohlschmidt N, Peltonen L, Kestila M. Mutation spectrum of Meckel syndrome genes: one group of syndromes or several distinct groups? *Hum Mutat* **30**, E813-830 (2009).

115. Harms FL, *et al.* Elsahy-Waters syndrome is caused by biallelic mutations in CDH11. *Am J Med Genet A* **176**, 477-482 (2018).

116. Picker-Minh S, *et al.* Phenotype variability of infantile-onset multisystem neurologic, endocrine, and pancreatic disease IMNEPD. *Orphanet J Rare Dis* **11**, 52 (2016).

117. Guo H, *et al.* NCKAP1 Disruptive Variants Lead to a Neurodevelopmental Disorder with Core Features of Autism. *Am J Hum Genet* **107**, 963-976 (2020).

118. Alazami AM, *et al.* A novel syndrome of Klippel-Feil anomaly, myopathy, and characteristic facies is linked to a null mutation in MYO18B. *J Med Genet* **52**, 400-404 (2015).

119. Shamseldin HE, *et al.* Identification of embryonic lethal genes in humans by autozygosity mapping and exome sequencing in consanguineous families. *Genome Biol* **16**, 116 (2015).

120. Alsultan A, Shamseldin HE, Osman ME, Aljabri M, Alkuraya FS. MYSM1 is mutated in a family with transient transfusion-dependent anemia, mild thrombocytopenia, and low NK- and B-cell counts. *Blood* **122**, 3844-3845 (2013).

121. Shaheen R, *et al.* Mutations in EOGT confirm the genetic heterogeneity of autosomal-recessive Adams-Oliver syndrome. *Am J Hum Genet* **92**, 598-604 (2013).

122. Shaheen R, *et al.* PUS7 mutations impair pseudouridylation in humans and cause intellectual disability and microcephaly. *Hum Genet* **138**, 231-239 (2019).

123. Monies D, *et al.* Lessons Learned from Large-Scale, First-Tier Clinical Exome Sequencing in a Highly Consanguineous Population. *Am J Hum Genet* **104**, 1182-1201 (2019).

124. Teebi AS, Farag TI. *Genetic disorders among Arab populations*. Oxford University Press (1997).

125. Chelban V, *et al.* Mutations in NKX6-2 Cause Progressive Spastic Ataxia and Hypomyelination. *Am J Hum Genet* **100**, 969-977 (2017).

126. Breuss MW, *et al.* Autosomal-Recessive Mutations in the tRNA Splicing Endonuclease Subunit TSEN15 Cause Pontocerebellar Hypoplasia and Progressive Microcephaly. *Am J Hum Genet* **99**, 228-235 (2016).

127. Alkhunaizi E, *et al.* Warsaw breakage syndrome: Further clinical and genetic delineation. *Am J Med Genet A* **176**, 2404-2418 (2018).

128. Shaheen R, Hashem A, Abdel-Salam GM, Al-Fadhli F, Ewida N, Alkuraya FS. Mutations in CIT, encoding citron rho-interacting serine/threonine kinase, cause severe primary microcephaly in humans. *Hum Genet* **135**, 1191-1197 (2016).

129. Patel N, *et al.* GZF1 Mutations Expand the Genetic Heterogeneity of Larsen Syndrome. *Am J Hum Genet* **100**, 831-836 (2017).

130. Khan AO, Alrashed M, Alkuraya FS. 'Cone dystrophy with supranormal rod response' in children. *Br J Ophthalmol* **96**, 422-426 (2012).

131. Shaheen R, *et al.* Mutations in SMG9, Encoding an Essential Component of Nonsense-Mediated Decay Machinery, Cause a Multiple Congenital Anomaly Syndrome in Humans and Mice. *Am J Hum Genet* **98**, 643-652 (2016).

132. Shtir C, *et al.* Exome-based case-control association study using extreme phenotype design reveals novel candidates with protective effect in diabetic retinopathy. *Hum Genet* **135**, 193-200 (2016).

133. Monies D, *et al.* Autozygosity reveals recessive mutations and novel mechanisms in dominant genes: implications in variant interpretation. *Genet Med* **19**, 1144-1150 (2017).

134. Albaqumi M, Alhabib FA, Shamseldin HE, Mohammed F, Alkuraya FS. A syndrome of congenital hyperinsulinism and rhabdomyolysis is caused by KCNJ11 mutation. *J Med Genet* **51**, 271-274 (2014).

135. Alazami AM, *et al.* C2orf37 mutational spectrum in Woodhouse-Sakati syndrome patients. *Clin Genet* **78**, 585-590 (2010).

136. Shamseldin HE, Yakulov TA, Hashem A, Walz G, Alkuraya FS. ANKS3 is mutated in a family with autosomal recessive laterality defect. *Hum Genet* **135**, 1233-1239 (2016).

137. Patel N, *et al.* Study of Mendelian forms of Crohn's disease in Saudi Arabia reveals novel risk loci and alleles. *Gut* **63**, 1831-1832 (2014).

138. Thomas AX, *et al.* ANKLE2-related microcephaly: A variable microcephaly syndrome resembling Zika infection. *Ann Clin Transl Neurol* **9**, 1276-1288 (2022).

139. Shamseldin HE, *et al.* An exome-first approach to aid in the diagnosis of primary ciliary dyskinesia. *Hum Genet* **139**, 1273-1283 (2020).

140. Alsaif HS, *et al.* ZNF668 deficiency causes a recognizable disorder of DNA damage repair. *Hum Genet* **140**, 1395-1401 (2021).

141. Rosenhahn E, *et al.* Bi-allelic loss-of-function variants in PPFIBP1 cause a neurodevelopmental disorder with microcephaly, epilepsy, and periventricular calcifications. *Am J Hum Genet* **109**, 1421-1435 (2022).

142. Al-Rashed M, *et al.* RP1 and retinitis pigmentosa: report of novel mutations and insight into mutational mechanism. *Br J Ophthalmol* **96**, 1018-1022 (2012).

143. Alharbi S, Alhashem A, Alkuraya F, Kashlan F, Tlili-Graiess K. Neuroimaging manifestations and genetic heterogeneity of Walker-Warburg syndrome in Saudi patients. *Brain Dev* **43**, 380-388 (2021).

144. Patel N, *et al.* A novel APC mutation defines a second locus for Cenani-Lenz syndrome. *J Med Genet* **52**, 317-321 (2015).

145. Patel N, *et al.* Mutations in ASPH cause facial dysmorphism, lens dislocation, anterior-segment abnormalities, and spontaneous filtering blebs, or Traboulsi syndrome. *Am J Hum Genet* **94**, 755-759 (2014).

146. Alzahrani F, Alshammari MJ, Alkuraya FS. Molecular pathogenesis of fibrochondrogenesis: is it really simple COL11A1 deficiency? *Gene* **511**, 480-481 (2012).

147. Shamseldin HE, *et al.* Mutation of the mitochondrial carrier SLC25A42 causes a novel form of mitochondrial myopathy in humans. *Hum Genet* **135**, 21-30 (2016).

148. Gueneau L, *et al.* KIAA1109 Variants Are Associated with a Severe Disorder of Brain Development and Arthrogryposis. *Am J Hum Genet* **102**, 116-132 (2018).

149. Alazami AM, *et al.* Loss of function mutation in LARP7, chaperone of 7SK ncRNA, causes a syndrome of facial dysmorphism, intellectual disability, and primordial dwarfism. *Hum Mutat* **33**, 1429-1434 (2012).

150. Carbonella A, *et al.* An autosomal recessive DNASE1L3-related autoimmune disease with unusual clinical presentation mimicking systemic lupus erythematosus. *Lupus* **26**, 768-772 (2017).

151. Faqeih EA, Almannai M, Saleh MM, AlWadei AH, Samman MM, Alkuraya FS. Phenotypic characterization of KCTD3-related developmental epileptic encephalopathy. *Clin Genet* **93**, 1081-1086 (2018).

152. Shamseldin HE, *et al.* The morbid genome of ciliopathies: an update. *Genet Med* **24**, 966 (2022).

153. Efthymiou S, *et al.* Biallelic mutations in neurofascin cause neurodevelopmental impairment and peripheral demyelination. *Brain* **142**, 2948-2964 (2019).

154. Maddirevula S, *et al.* GWAS signals revisited using human knockouts. *Genet Med* **20**, 64-68 (2018).

155. Altuame FD*, et al.* The natural history of infantile neuroaxonal dystrophy. *Orphanet J Rare Dis* **15**, 109 (2020).

156. Ali Alghamdi M*, et al.* Molecular autopsy by proxy in preconception counseling. *Clin Genet* **100**, 678-691 (2021).

157. Shaheen R, Al Tala S, Almoisheer A, Alkuraya FS. Mutation in PLK4, encoding a master regulator of centriole formation, defines a novel locus for primordial dwarfism. *J Med Genet* **51**, 814-816 (2014).

158. Almannai M*, et al.* Expanding the phenotype of SLC25A42-associated mitochondrial encephalomyopathy. *Clin Genet* **93**, 1097-1102 (2018).

159. Janecke AR*, et al.* Pathogenic STX3 variants affecting the retinal and intestinal transcripts cause an early-onset severe retinal dystrophy in microvillus inclusion disease subjects. *Hum Genet* **140**, 1143-1156 (2021).

160. Alazami AM*, et al.* TLE6 mutation causes the earliest known human embryonic lethality. *Genome Biol* **16**, 240 (2015).

161. Suleiman J, Allingham-Hawkins D, Hashem M, Shamseldin HE, Alkuraya FS, El-Hattab AW. WDR45B-related intellectual disability, spastic quadriplegia, epilepsy, and cerebral hypoplasia: A consistent neurodevelopmental syndrome. *Clin Genet* **93**, 360-364 (2018).

162. Shamseldin HE*, et al.* Novel copy number variants and major limb reduction malformation: Report of three cases. *Am J Med Genet A* **170A**, 1245-1250 (2016).

163. Shaheen R*, et al.* Clinical, biochemical and molecular characterization of peroxisomal diseases in Arabs. *Clin Genet* **79**, 60-70 (2011).

164. Sewairi W, Assiri A, Patel N, Alhashem A, Alkuraya FS. Distal acroosteolysis, poikiloderma and joint stiffness: a novel laminopathy? *Eur J Hum Genet* **24**, 1220-1222 (2016).

165. Shaheen R*, et al.* Mutation in WDR4 impairs tRNA m(7)G46 methylation and causes a distinct form of microcephalic primordial dwarfism. *Genome Biol* **16**, 210 (2015).

166. Anazi S*, et al.* Correction to: Expanding the genetic heterogeneity of intellectual disability. *Hum Genet* **137**, 105-109 (2018).

167. Shamseldin H*, et al.* RTTN Mutations Cause Primary Microcephaly and Primordial Dwarfism in Humans. *Am J Hum Genet* **97**, 862-868 (2015).

168. Shamseldin HE*, et al.* KDF1, encoding keratinocyte differentiation factor 1, is mutated in a multigenerational family with ectodermal dysplasia. *Hum Genet* **136**, 99-105 (2017).

169. Shamseldin HE*, et al.* NUP214 deficiency causes severe encephalopathy and microcephaly in humans. *Hum Genet* **138**, 221-229 (2019).

170. Wiessner M*, et al.* Biallelic variants in HPDL cause pure and complicated hereditary spastic paraplegia. *Brain* **144**, 1422-1434 (2021).

171. Shamseldin HE*, et al.* Mitochondrial "dysmorphology" in variant classification. *Hum Genet* **141**, 55-64 (2022).

172. Shamseldin HE, Aldeeri A, Babay Z, Alsultan A, Hashem M, Alkuraya FS. A lethal phenotype associated with tissue plasminogen deficiency in humans. *Hum Genet* **135**, 1209-1211 (2016).

173. Seidahmed MZ*, et al.* Report of a case of Raine syndrome and literature review. *Am J Med Genet A* **167A**, 2394-2398 (2015).

174. Shamseldin HE*, et al.* GNB5 mutation causes a novel neuropsychiatric disorder featuring attention deficit hyperactivity disorder, severely impaired language development and normal cognition. *Genome Biol* **17**, 195 (2016).

175. Nowilaty SR, Khan AO, Aldahmesh MA, Tabbara KF, Al-Amri A, Alkuraya FS. Biometric and molecular characterization of clinically diagnosed posterior microphthalmos. *Am J Ophthalmol* **155**, 361-372 e367 (2013).

176. Kruer MC*, et al.* C19orf12 mutation leads to a pallido-pyramidal syndrome. *Gene* **537**, 352-356 (2014).

177. Aldahmesh MA*, et al.* Posterior microphthalmos as a genetically heterogeneous condition that can be allelic to nanophthalmos. *Arch Ophthalmol* **129**, 805-807 (2011).

178. Seidahmed MZ*, et al.* Gonadal mosaicism for ACTA1 gene masquerading as autosomal recessive nemaline myopathy. *Am J Med Genet A* **170**, 2219-2221 (2016).

179. Shamseldin HE, Faqeih E, Alasmari A, Zaki MS, Gleeson JG, Alkuraya FS. Mutations in UNC80, Encoding Part of the UNC79-UNC80-NALCN Channel Complex, Cause Autosomal-Recessive Severe Infantile Encephalopathy. *Am J Hum Genet* **98**, 210-215 (2016).

180. Shamseldin HE, Bennett AH, Alfadhel M, Gupta V, Alkuraya FS. GOLGA2, encoding a master regulator of golgi apparatus, is mutated in a patient with a neuromuscular disorder. *Hum Genet* **135**, 245-251 (2016).

181. Maddirevula S*, et al.* Further delineation of HIDEA syndrome. *Am J Med Genet A* **182**, 2999-3006 (2020).

182. Shaheen R, Al Tala S, Ewida N, Abouelhoda M, Alkuraya FS. Epileptic encephalopathy with continuous spike-and-wave during sleep maps to a homozygous truncating mutation in AMPA receptor component FRRS1L. *Clin Genet* **90**, 282-283 (2016).

183. van der Knoop MM*, et al.* Biallelic ADAM22 pathogenic variants cause progressive encephalopathy and infantile-onset refractory epilepsy. *Brain* **145**, 2301-2312 (2022).

184. Broly M*, et al.* THUMPD1 bi-allelic variants cause loss of tRNA acetylation and a syndromic neurodevelopmental disorder. *Am J Hum Genet* **109**, 587-600 (2022).

185. Zha C*, et al.* Biallelic variants in the small optic lobe calpain CAPN15 are associated with congenital eye anomalies, deafness and other neurodevelopmental deficits. *Hum Mol Genet* **29**, 3054-3063 (2020).

186. Van De Weghe JC*, et al.* Mutations in ARMC9, which Encodes a Basal Body Protein, Cause Joubert Syndrome in Humans and Ciliopathy Phenotypes in Zebrafish. *Am J Hum Genet* **101**, 23-36 (2017).

187. Aldinger KA*, et al.* Redefining the Etiologic Landscape of Cerebellar Malformations. *Am J Hum Genet* **105**, 606-615 (2019).

188. Shaheen R*, et al.* A homozygous truncating mutation in PUS3 expands the role of tRNA modification in normal cognition. *Hum Genet* **135**, 707-713 (2016).

189. Altuwaijri N*, et al.* Further delineation of SMG9-related heart and brain malformation syndrome. *Am J Med Genet A* **185**, 1624-1630 (2021).

190. Alazami AM*, et al.* Molecular characterization of Joubert syndrome in Saudi Arabia. *Hum Mutat* **33**, 1423-1428 (2012).

191. Al-Abdi L*, et al.* CNP deficiency causes severe hypomyelinating leukodystrophy in humans. *Hum Genet* **139**, 615-622 (2020).

192. Seidahmed MZ, Maddirevula S, Miqdad AM, Al Faifi A, Al Samadi A, Alkuraya FS. Confirming the involvement of PIEZO2 in the etiology of Marden-Walker syndrome. *Am J Med Genet A* **185**, 945-948 (2021).

193. Moawia A*, et al.* Mutations of KIF14 cause primary microcephaly by impairing cytokinesis. *Ann Neurol* **82**, 562-577 (2017).

194. Al Mutairi F, Shamseldin HE, Alfadhel M, Rodenburg RJ, Alkuraya FS. A lethal neonatal phenotype of mitochondrial short-chain enoyl-CoA hydratase-1 deficiency. *Clin Genet* **91**, 629-633 (2017).

195. Maddirevula S*, et al.* A genomics approach to females with infertility and recurrent pregnancy loss. *Hum Genet* **139**, 605-613 (2020).

196. Stephen J*, et al.* Bi-allelic TMEM94 Truncating Variants Are Associated with Neurodevelopmental Delay, Congenital Heart Defects, and Distinct Facial Dysmorphism. *Am J Hum Genet* **103**, 948-967 (2018).

197. Melo US*, et al.* Biallelic UBE4A loss-of-function variants cause intellectual disability and global developmental delay. *Genet Med* **23**, 661-668 (2021).

198. Maddirevula S*, et al.* Female Infertility Caused by Mutations in the Oocyte-Specific Translational Repressor PATL2. *Am J Hum Genet* **101**, 603-608 (2017).

199. Shamseldin HE*, et al.* A null mutation in MICU2 causes abnormal mitochondrial calcium homeostasis and a severe neurodevelopmental disorder. *Brain* **140**, 2806-2813 (2017).

200. Shaheen R, Al-Owain M, Sakati N, Alzayed ZS, Alkuraya FS. FKBP10 and Bruck syndrome: phenotypic heterogeneity or call for reclassification? *Am J Hum Genet* **87**, 306-307; author reply 308 (2010).

201. Maddirevula S, Abanemai M, Alkuraya FS. Human knockouts of PLA2G4A phenocopy NSAID-induced gastrointestinal and renal toxicity. *Gut* **65**, 1575-1577 (2016).

202. Alzahrani F, Alkeraye S, Alkuraya FS. The alternatively spliced exon of COL5A1 is mutated in autosomal recessive classical Ehlers-Danlos syndrome. *Clin Genet* **93**, 936-937 (2018).

203. Shaheen R*, et al.* Biallelic Mutations in Tetratricopeptide Repeat Domain 26 (Intraflagellar Transport 56) Cause Severe Biliary Ciliopathy in Humans. *Hepatology* **71**, 2067-2079 (2020).

204. Alghamdi M*, et al.* A Novel TBX1 Variant Causing Hypoparathyroidism and Deafness. *J Endocr Soc* **4**, bvz028 (2020).

205. Altawil L, Alshihry H, Alfaraidi H, Alhashem A, Alhumidi A, Alkuraya FS. Progressive symmetrical erythrokeratoderma manifesting as harlequin-like ichthyosis with severe thrombocytopenia secondary to a homozygous 3-ketodihydrosphingosine reductase mutation. *JAAD Case Rep* **14**, 55-58 (2021).

206. Neuray C*, et al.* Early-infantile onset epilepsy and developmental delay caused by bi-allelic GAD1 variants. *Brain* **143**, 2388-2397 (2020).

207. Maddirevula S, *et al.* Identification of novel loci for pediatric cholestatic liver disease defined by KIF12, PPM1F, USP53, LSR, and WDR83OS pathogenic variants. *Genet Med* **21**, 1164-1172 (2019).

208. Alsahan N, Alkuraya FS. Confirming TBC1D32-related ciliopathy in humans. *Am J Med Genet A* **182**, 1985-1987 (2020).

209. Monies D, Vagbo CB, Al-Owain M, Alhomaidi S, Alkuraya FS. Recessive Truncating Mutations in ALKBH8 Cause Intellectual Disability and Severe Impairment of Wobble Uridine Modification. *Am J Hum Genet* **104**, 1202-1209 (2019).

210. Aldahmesh MA*, et al.* Recessive mutations in ELOVL4 cause ichthyosis, intellectual disability, and spastic quadriplegia. *Am J Hum Genet* **89**, 745-750 (2011).

211. Broeks MH*, et al.* MDH1 deficiency is a metabolic disorder of the malate-aspartate shuttle associated with early onset severe encephalopathy. *Hum Genet* **138**, 1247-1257 (2019).

212. Palmer EE*, et al.* De Novo Variants Disrupting the HX Repeat Motif of ATN1 Cause a Recognizable Non-Progressive Neurocognitive Syndrome. *Am J Hum Genet* **104**, 542-552 (2019).

213. Kalantari S*, et al.* Expanding the KIF4A-associated phenotype. *Am J Med Genet A* **185**, 3728-3739 (2021).

214. Palmer EE*, et al.* CHEDDA syndrome is an underrecognized neurodevelopmental disorder with a highly restricted ATN1 mutation spectrum. *Clin Genet* **100**, 468-477 (2021).

215. Hengel H*, et al.* Loss-of-function mutations in UDP-Glucose 6-Dehydrogenase cause recessive developmental epileptic encephalopathy. *Nat Commun* **11**, 595 (2020).

216. Chivukula RR*, et al.* A human ciliopathy reveals essential functions for NEK10 in airway mucociliary clearance. *Nat Med* **26**, 244-251 (2020).

217. Monies D, *et al.* Lessons Learned from Large-Scale, First-Tier Clinical Exome Sequencing in a Highly Consanguineous Population. *Am J Hum Genet* **104**, 1182-1201 (2019).

218. Alkanderi S*, et al.* ARL3 Mutations Cause Joubert Syndrome by Disrupting Ciliary Protein Composition. *Am J Hum Genet* **103**, 612-620 (2018).

219. Schanzer A*, et al.* Mutations in HID1 Cause Syndromic Infantile Encephalopathy and Hypopituitarism. *Ann Neurol* **90**, 143-158 (2021).

220. Alosaimi MF*, et al.* Immunodeficiency and EBV-induced lymphoproliferation caused by 4-1BB deficiency. *J Allergy Clin Immunol* **144**, 574-583 e575 (2019).

221. Patel N, Nabil A, Alshammari M, Alkuraya FS. Hoarse voice in children as the presenting feature of ECM1-related lipoid proteinosis. *Am J Med Genet A* **185**, 3924-3925 (2021).

222. Maddirevula S*, et al.* Insight into ALKBH8-related intellectual developmental disability based on the first pathogenic missense variant. *Hum Genet* **141**, 209-215 (2022).

223. Murakami Y*, et al.* Mutations in PIGB Cause an Inherited GPI Biosynthesis Defect with an Axonal Neuropathy and Metabolic Abnormality in Severe Cases. *Am J Hum Genet* **105**, 384-394 (2019).

224. Altuame FD*, et al.* PLXNA2 as a candidate gene in patients with intellectual disability. *Am J Med Genet A* **185**, 3859-3865 (2021).

225. Lin SJ*, et al.* Biallelic variants in KARS1 are associated with neurodevelopmental disorders and hearing loss recapitulated by the knockout zebrafish. *Genet Med* **23**, 1933-1943 (2021).

226. Thomas Q*, et al.* Haploinsufficiency of ARFGEF1 is associated with developmental delay, intellectual disability, and epilepsy with variable expressivity. *Genet Med* **23**, 1901-1911 (2021).

227. Pottie L*, et al.* Bi-allelic premature truncating variants in LTBP1 cause cutis laxa syndrome. *Am J Hum Genet* **108**, 2386-2388 (2021).

228. Shaheen R*, et al.* A TCTN2 mutation defines a novel Meckel Gruber syndrome locus. *Hum Mutat* **32**, 573-578 (2011).

229. Alanzi T, Alhashem A, Dagriri K, Alzahrani F, Alkuraya FS. A de novo splicing variant supports the candidacy of TLL1 in ASD pathogenesis. *Eur J Hum Genet* **28**, 525-528 (2020).

230. Altawil L, Alshihry H, Ahmed H, Shamseldin HE, Alkuraya F. Vitamin B12 deficiency secondary to cobalamin F deficiency simulating dyskeratosis congenita. *JAAD Case Rep* **6**, 882-885 (2020).

231. Suleiman J*, et al.* Homozygous loss-of-function variants of TASP1, a gene encoding an activator of the histone methyltransferases KMT2A and KMT2D, cause a syndrome of developmental delay, happy demeanor, distinctive facial features, and congenital anomalies. *Hum Mutat* **40**, 1985-1992 (2019).

232. Al-Hamed MH*, et al.* A null founder variant in NPNT, encoding nephronectin, causes autosomal recessive renal agenesis. *Clin Genet* **102**, 61-65 (2022).

233. Alzahrani F*, et al.* Recessive, Deleterious Variants in SMG8 Expand the Role of Nonsense-Mediated Decay in Developmental Disorders in Humans. *Am J Hum Genet* **107**, 1178-1185 (2020).

234. Alhathal N*, et al.* A genomics approach to male infertility. *Genet Med* **22**, 1967-1975 (2020).

235. Wallmeier J*, et al.* Mutations in TP73 cause impaired mucociliary clearance and lissencephaly. *Am J Hum Genet* **108**, 1318-1329 (2021).

236. Alsaif HS*, et al.* Homozygous Loss-of-Function Mutations in AP1B1, Encoding Beta-1 Subunit of Adaptor-Related Protein Complex 1, Cause MEDNIK-like Syndrome. *Am J Hum Genet* **105**, 1016-1022 (2019).

237. Lentini JM, Alsaif HS, Faqeih E, Alkuraya FS, Fu D. DALRD3 encodes a protein mutated in epileptic encephalopathy that targets arginine tRNAs for 3-methylcytosine modification. *Nat Commun* **11**, 2510 (2020).

238. Morrison J*, et al.* Missense NAA20 variants impairing the NatB protein N-terminal acetyltransferase cause autosomal recessive developmental delay, intellectual disability, and microcephaly. *Genet Med* **23**, 2213-2218 (2021).

239. Alazami AM*, et al.* Congenital disorder of glycosylation IIa: the trouble with diagnosing a dysmorphic inborn error of metabolism. *Am J Med Genet A* **158A**, 245-246 (2012).

240. Almaghlouth IA, Mohamed JY, Al-Amoudi M, Al-Ahaidib L, Al-Odaib A, Alkuraya FS. 5-Oxoprolinase deficiency: report of the first human OPLAH mutation. *Clin Genet* **82**, 193-196 (2012).

241. Aldahmesh MA, Khan AO, Hijazi H, Alkuraya FS. Mutations in ALDH1A3 cause microphthalmia. *Clin Genet* **84**, 128-131 (2013).

242. Scala M*, et al.* Biallelic MFSD2A variants associated with congenital microcephaly, developmental delay, and recognizable neuroimaging features. *Eur J Hum Genet* **28**, 1509-1519 (2020).

243. Hengel H*, et al.* Bi-allelic loss-of-function variants in BCAS3 cause a syndromic neurodevelopmental disorder. *Am J Hum Genet* **108**, 1069-1082 (2021).

244. Chen YH*, et al.* Absence of GP130 cytokine receptor signaling causes extended Stuve-Wiedemann syndrome. *J Exp Med* **217**, (2020).

245. Averdunk L*, et al.* Correction to: The recurrent missense mutation p.(Arg367Trp) in YARS1 causes a distinct neurodevelopmental phenotype. *J Mol Med (Berl)* **99**, 1769-1770 (2021).

246. Marafi D*, et al.* Biallelic variants in SLC38A3 encoding a glutamine transporter cause epileptic encephalopathy. *Brain* **145**, 909-924 (2022).

247. Alazami AM, Hijazi H, Kentab AY, Alkuraya FS. NECAP1 loss of function leads to a severe infantile epileptic encephalopathy. *J Med Genet* **51**, 224-228 (2014).

248. Seidahmed MZ*, et al.* Recessive mutations in SCYL2 cause a novel syndromic form of arthrogryposis in humans. *Hum Genet* **139**, 513-519 (2020).

249. Alazami AM, Maddirevula S, Seidahmed MZ, Albhlal LA, Alkuraya FS. A novel ISLR2-linked autosomal recessive syndrome of congenital hydrocephalus, arthrogryposis and abdominal distension. *Hum Genet* **138**, 105-107 (2019).

250. Collier JJ*, et al.* Developmental Consequences of Defective ATG7-Mediated Autophagy in Humans. *N Engl J Med* **384**, 2406-2417 (2021).

251. Wallmeier J*, et al.* Mutations in CCNO result in congenital mucociliary clearance disorder with reduced generation of multiple motile cilia. *Nat Genet* **46**, 646-651 (2014).

252. Alsaif HS*, et al.* MYH1 is a candidate gene for recurrent rhabdomyolysis in humans. *Am J Med Genet A* **185**, 2131-2135 (2021).

253. AlAbdi L*, et al.* Loss-of-function variants in MYCBP2 cause neurobehavioural phenotypes and corpus callosum defects. *Brain* **146**, 1373-1387 (2023).

254. Almannai M*, et al.* KIF26A is mutated in the syndrome of congenital hydrocephalus with megacolon. *Hum Genet* **142**, 399-405 (2023).

255. Maddirevula S*, et al.* ASTL is mutated in female infertility. *Hum Genet* **141**, 49-54 (2022).

256. Shankar SP*, et al.* A novel DPH5-related diphthamide-deficiency syndrome causing embryonic lethality or profound neurodevelopmental disorder. *Genet Med* **24**, 1567-1582 (2022).

257. Aldahmesh MA*, et al.* Identification of ADAMTS18 as a gene mutated in Knobloch syndrome. *J Med Genet* **48**, 597-601 (2011).

258. Magrinelli F, *et al.* Biallelic Loss-of-Function NDUFA12 Variants Cause a Wide Phenotypic Spectrum from Leigh/Leigh-Like Syndrome to Isolated Optic Atrophy. *Mov Disord Clin Pract* **9**, 218-228 (2022).

259. Alkuraya FS. Arthrogryposis, perthes disease, and upward gaze palsy: a novel autosomal recessive syndromic form of arthrogryposis. *Am J Med Genet A* **155A**, 297-300 (2011).

260. Sheppard SE, *et al.* Expanding the genotypic and phenotypic spectrum in a diverse cohort of 104 individuals with Wiedemann-Steiner syndrome. *Am J Med Genet A* **185**, 1649-1665 (2021).

261. Alharby E, *et al.* Clinical, molecular, and biochemical delineation of asparagine synthetase deficiency in Saudi cohort. *Genet Med* **22**, 2071-2080 (2020).

262. Al-Qattan MM, Maddirevula S, Alkuraya FS. A de novo TBX3 mutation presenting as dorsalization of the little fingers: A forme fruste phenotype of ulnar-mammary syndrome. *Eur J Med Genet* **63**, 103615 (2020).

263. Khouj E, Bohlega S, Alkuraya FS. Cerebrotendinous xanthomatosis: A candidate for ACMG list of secondary findings? *Clin Genet* **103**, 125-126 (2023).

264. Palmer EE, *et al.* De Novo Variants Disrupting the HX Repeat Motif of ATN1 Cause a Recognizable Non-progressive Neurocognitive Syndrome. *Am J Hum Genet* **104**, 778 (2019).

265. Altuame FD, *et al.* Further delineation of MYO18B-related autosomal recessive Klippel-Feil syndrome with myopathy and facial dysmorphism. *Am J Med Genet A* **185**, 370-376 (2021).

266. Reiff RE, *et al.* METTL23, a transcriptional partner of GABPA, is essential for human cognition. *Hum Mol Genet* **23**, 3456-3466 (2014).

267. Mericq V, *et al.* Biallelic POC1A variants cause syndromic severe insulin resistance with muscle cramps. *Eur J Endocrinol* **186**, 543-552 (2022).

268. Alhashem AM, *et al.* Crisponi/CISS1 syndrome: A case series. *Am J Med Genet A* **170A**, 1236-1241 (2016).

269. Patel N, *et al.* Confirming the recessive inheritance of PERP-related erythrokeratoderma. *Clin Genet* **97**, 661-665 (2020).

270. Al-Owain M, *et al.* A case of de Barsy syndrome with a severe eye phenotype. *Am J Med Genet A* **158A**, 2364-2366 (2012).

271. Al-Qattan SM, *et al.* The clinical utility of molecular karyotyping for neurocognitive phenotypes in a consanguineous population. *Genet Med* **17**, 719-725 (2015).

272. Bertoli-Avella AM, *et al.* Combining exome/genome sequencing with data repository analysis reveals novel gene-disease associations for a wide range of genetic disorders. *Genet Med* **23**, 1551-1568 (2021).

273. Alsum Z, Abu Safieh L, Nygren AO, Al-Hamed MA, Alkuraya FS. Methylation-specific multiplex-ligation-dependent probe amplification as a rapid molecular diagnostic tool for pseudohypoparathyroidism type 1b. *Genet Test Mol Biomarkers* **14**, 135-139 (2010).

274. Suleiman J, Al Hashem AM, Tabarki B, Al-Thihli K, Bi W, El-Hattab AW. PPP1R21 homozygous null variants associated with developmental delay, muscle weakness, distinctive facial features, and brain abnormalities. *Clin Genet* **94**, 351-355 (2018).

275. Lachke SA, *et al.* Mutations in the RNA granule component TDRD7 cause cataract and glaucoma. *Science* **331**, 1571-1576 (2011).

276. Shamseldin HE, *et al.* PRSS8, encoding prostasin, is mutated in patients with autosomal recessive ichthyosis. *Hum Genet* **142**, 477-482 (2023).

277. Anazi S, *et al.* A null mutation in TNIK defines a novel locus for intellectual disability. *Hum Genet* **135**, 773-778 (2016).

278. Wagner M, *et al.* Bi-allelic Variants in RALGAPA1 Cause Profound Neurodevelopmental Disability, Muscular Hypotonia, Infantile Spasms, and Feeding Abnormalities. *Am J Hum Genet* **106**, 246-255 (2020).

279. McQuaid ME, *et al.* Hypomorphic GINS3 variants alter DNA replication and cause Meier-Gorlin syndrome. *JCI Insight* **7**, (2022).

280. Shaheen R, Ansari S, Mardawi EA, Alshammari MJ, Alkuraya FS. Mutations in TMEM231 cause Meckel-Gruber syndrome. *J Med Genet* **50**, 160-162 (2013).

281. Khan AO, Aldahmesh MA, Alkuraya FS. Phenotypes of Recessive Pediatric Cataract in a Cohort of Children with Identified Homozygous Gene Mutations (An American Ophthalmological Society Thesis). *Trans Am Ophthalmol Soc* **113**, T7 (2015).

282. Drissi I, *et al.* Mutations in phospholipase C eta-1 (PLCH1) are associated with holoprosencephaly. *J Med Genet* **59**, 358-365 (2022).

283. Iqbal M, *et al.* Biallelic variants in PCDHGC4 cause a novel neurodevelopmental syndrome with progressive microcephaly, seizures, and joint anomalies. *Genet Med* **23**, 2138-2149 (2021).

284. Choufani S, *et al.* An HNRNPK-specific DNA methylation signature makes sense of missense variants and expands the phenotypic spectrum of Au-Kline syndrome. *Am J Hum Genet* **109**, 1867-1884 (2022).

285. Patel N, *et al.* Expanding the clinical spectrum and allelic heterogeneity in van den Ende-Gupta syndrome. *Clin Genet* **85**, 492-494 (2014).

286. Ramond F, *et al.* Clustered variants in the 5' coding region of TRA2B cause a distinctive neurodevelopmental syndrome. *Genet Med* **25**, 100003 (2023).

287. Al-Hamed MH, *et al.* Bialleleic PKD1 mutations underlie early-onset autosomal dominant polycystic kidney disease in Saudi Arabian families. *Pediatr Nephrol* **34**, 1615-1623 (2019).

288. Webb EA, *et al.* ARNT2 mutation causes hypopituitarism, post-natal microcephaly, visual and renal anomalies. *Brain* **136**, 3096-3105 (2013).

289. Khodadadi H, *et al.* PTRHD1 (C2orf79) mutations lead to autosomal-recessive intellectual disability and parkinsonism. *Mov Disord* **32**, 287-291 (2017).

290. Fakhro KA, *et al.* Point of Care Exome Sequencing Reveals Allelic and Phenotypic Heterogeneity Underlying Mendelian disease in Qatar. *Hum Mol Genet* **28**, 3970-3981 (2019).

291. Nakhro K, *et al.* SET binding factor 1 (SBF1) mutation causes Charcot-Marie-Tooth disease type 4B3. *Neurology* **81**, 165-173 (2013).

292. Lin Z, *et al.* Stabilizing mutations of KLHL24 ubiquitin ligase cause loss of keratin 14 and human skin fragility. *Nat Genet* **48**, 1508-1516 (2016).

293. Monies D, *et al.* The landscape of genetic diseases in Saudi Arabia based on the first 1000 diagnostic panels and exomes. *Hum Genet* **136**, 921-939 (2017).

294. Kriegova E, *et al.* Whole-genome optical mapping of bone-marrow myeloma cells reveals association of extramedullary multiple myeloma with chromosome 1 abnormalities. *Sci Rep* **11**, 14671 (2021).
