# Supplementary material for: Diagnostic implications of pitfalls in causal variant identification based on 4577 molecularly characterized families
Source: Nat Commun. 2023 Aug 29;14:5269. doi: 10.1038/s41467-023-40909-3 (PMC10465531; doi:10.1038/s41467-023-40909-3)
Supplement: Supplementary file 3 — Description of Additional Supplementary Files [file 41467_2023_40909_MOESM3_ESM.pdf]

Description of Additional Supplementary Files

File Name: Supplementary Data 1

Description: List of all families with challenges included in this study.

File Name: Supplementary Data 2

Description: List of all families with phenotypic heterogeneity.

File Name: Supplementary Data 3

Description: List of all families with phenotypic expansion.

File Name: Supplementary Data 4

Description: List of all families with novel allelic disorders.

File Name: Supplementary Data 5

Description: List of all families with blended phenotypes.

File Name: Supplementary Data 6

Description: List of all families with erroneous clinical labels.

File Name: Supplementary Data 7

Description: List of all families with novel disease genes.

File Name: Supplementary Data 8

Description: List of all families where the gene expressed a phenotype that was incompatible with that observed in the animal model.

File Name: Supplementary Data 9

Description: List of all families with known genes, novel mutation mechanisms.

File Name: Supplementary Data 10

Description: List of all families with tentative transcript deleterious variants.

File Name: Supplementary Data 11

Description: List of all families with causal variants that have allele frequency above cut-off.

File Name: Supplementary Data 12

Description: List of all families with variants with challenging in silico prediction.

File Name: Supplementary Data 13

Description: Cases with penetrance challenges.

File Name: Supplementary Data 14

Description: List of all families where another variant caused distraction from the causal variant.

File Name: Supplementary Data 15

Description: List of all families where a potentially dispensable gene was identified.

File Name: Supplementary Data 16

Description: List of all families solved with deep intronic variants that are typically not discoverable by exome sequencing.

File Name: Supplementary Data 17

Description: List of all families solved with variants in regulatory elements.

File Name: Supplementary Data 18

Description: List of all families solved with repeat expansions.

File Name: Supplementary Data 19

Description: List of all families solved with genomic rearrangements.

File Name: Supplementary Data 20

Description: List of all families with pedigree-related challenges.

File Name: Supplementary Data 21

Description: List of all families with positional mapping-related challenges.

File Name: Supplementary Data 22

Description: List of all families with negative clinical exome or genome and breakdown of why the causal variants were missed.

File Name: Supplementary Data 23

Description: List of founder variants.

File Name: Supplementary Data 24

Description: List of novel gene-disease assertions that were corroborated by international collaborations.
