# Supplementary material for: Diagnostic implications of pitfalls in causal variant identification based on 4577 molecularly characterized families
Source: Nat Commun. 2023 Aug 29;14:5269. doi: 10.1038/s41467-023-40909-3 (PMC10465531; doi:10.1038/s41467-023-40909-3)
Supplement: Supplementary file 28 — Reporting Summary [file 41467_2023_40909_MOESM28_ESM.pdf]

nature portfolio

Corresponding author(s): Fowzan Alkuraya

Last updated by author(s): Mar 13, 2023

Reporting Summary

Nature Portfolio wishes to improve the reproducibility of the work that we publish. This form provides structure for consistency and transparency in reporting. For further information on Nature Portfolio policies, see our Editorial Policies and the Editorial Policy Checklist.

Statistics

For all statistical analyses, confirm that the following items are present in the figure legend, table legend, main text, or Methods section.

n/a	Confirmed	
☐	☒	The exact sample size (n) for each experimental group/condition, given as a discrete number and unit of measurement
☐	☒	A statement on whether measurements were taken from distinct samples or whether the same sample was measured repeatedly
☒	☐	The statistical test(s) used AND whether they are one- or two-sided<br>*Only common tests should be described solely by name; describe more complex techniques in the Methods section.*
☒	☐	A description of all covariates tested
☒	☐	A description of any assumptions or corrections, such as tests of normality and adjustment for multiple comparisons
☐	☒	A full description of the statistical parameters including central tendency (e.g. means) or other basic estimates (e.g. regression coefficient) AND variation (e.g. standard deviation) or associated estimates of uncertainty (e.g. confidence intervals)
☒	☐	For null hypothesis testing, the test statistic (e.g. F, t, r) with confidence intervals, effect sizes, degrees of freedom and P value noted<br>*Give P values as exact values whenever suitable.*
☒	☐	For Bayesian analysis, information on the choice of priors and Markov chain Monte Carlo settings
☒	☐	For hierarchical and complex designs, identification of the appropriate level for tests and full reporting of outcomes
☒	☐	Estimates of effect sizes (e.g. Cohen's d, Pearson's r), indicating how they were calculated

Our web collection on statistics for biologists contains articles on many of the points above.

Software and code

Policy information about availability of computer code

Data collection	Bionano software (v3.7)
Data analysis	ConsCal (v.2), Bionano software (v3.7), and GraphPad Prism (8.4.3)

For manuscripts utilizing custom algorithms or software that are central to the research but not yet described in published literature, software must be made available to editors and reviewers. We strongly encourage code deposition in a community repository (e.g. GitHub). See the Nature Portfolio guidelines for submitting code & software for further information.

Data

Policy information about availability of data

All manuscripts must include a data availability statement. This statement should provide the following information, where applicable:

- Accession codes, unique identifiers, or web links for publicly available datasets
- A description of any restrictions on data availability
- For clinical datasets or third party data, please ensure that the statement adheres to our policy

Due to local privacy laws and privileged human information, all requests are subject to prior approval from the local IRB, UCSC hg19 was used

Human research participants

Policy information about studies involving human research participants and Sex and Gender in Research.

Reporting on sex and gender	sex- and gender-based analyses were not performed because these are not relevant to the research question we set out to address i.e. what are the factors that render exome sequencing negative in patients with genetic diseases. Sex and/or gender of participants was determined based on self-report by the patients or their guardians.
Population characteristics	NA
Recruitment	Patients with a suspected Mendelian disease were recruited and consented under different IRB-approved projects. Recruitment is initiated by a referring physician and there is no recruitment bias that would impact the study
Ethics oversight	KFSHRC (RAC#2070023, 2080006, 2121053, 2170028, 2200030, 2080033, 2210029, 2080051, and 2200030)

Note that full information on the approval of the study protocol must also be provided in the manuscript.

Field-specific reporting

Please select the one below that is the best fit for your research. If you are not sure, read the appropriate sections before making your selection.

☒ Life sciences ☐ Behavioural & social sciences ☐ Ecological, evolutionary & environmental sciences

For a reference copy of the document with all sections, see nature.com/documents/nr-reporting-summary-flat.pdf

Life sciences study design

All studies must disclose on these points even when the disclosure is negative.

Sample size	No statistical methods were used to predetermine the cohort size. Sample size we referred to in the manuscript were simply the entire research cohort recruited over the past 15 years
Data exclusions	No data were excluded from the analyses
Replication	All data generated from experimental measures were replicated at least twice to ensure replication success
Randomization	Since we are not referring to particular conditions, randomization is not necessary.
Blinding	Since we are not referring to particular conditions, randomization is not necessary

Reporting for specific materials, systems and methods

We require information from authors about some types of materials, experimental systems and methods used in many studies. Here, indicate whether each material, system or method listed is relevant to your study. If you are not sure if a list item applies to your research, read the appropriate section before selecting a response.

Materials & experimental systems

n/a	Involved in the study	
☒	☐	Antibodies
☐	☒	Eukaryotic cell lines
☒	☐	Palaeontology and archaeology
☒	☐	Animals and other organisms
☐	☒	Clinical data
☒	☐	Dual use research of concern

Methods

n/a	Involved in the study	
☒	☐	ChIP-seq
☒	☐	Flow cytometry
☐	☒	MRI-based neuroimaging

Eukaryotic cell lines

Policy information about cell lines and Sex and Gender in Research

Cell line source(s)	Human derived lymphoblastoid and fibroblast cell lines

Authentication	The cell lines were authenticated by checking that the variant identified from the whole blood sample is also present in the DNA extracted from the cell line
Mycoplasma contamination	All cell lines were tested for mycoplasma contamination
Commonly misidentified lines (See ICLAC register)	NA

Clinical data

Policy information about clinical studies
All manuscripts should comply with the ICMJE guidelines for publication of clinical research and a completed CONSORT checklist must be included with all submissions.

Clinical trial registration	NA
Study protocol	NA
Data collection	NA
Outcomes	NA

Magnetic resonance imaging

Experimental design

Design type	NA
Design specifications	NA
Behavioral performance measures	NA

Acquisition

Imaging type(s)	NA
Field strength	NA
Sequence & imaging parameters	NA
Area of acquisition	NA

Diffusion MRI ☐ Used ☒ Not used

Preprocessing

Preprocessing software	NA
Normalization	NA
Normalization template	NA
Noise and artifact removal	NA
Volume censoring	NA

Statistical modeling & inference

Model type and settings	NA
Effect(s) tested	NA

Specify type of analysis: ☒ Whole brain ☐ ROI-based ☐ Both

Statistic type for inference (See Eklund et al. 2016)	NA
Correction	NA

Models & analysis

n/a	Involved in the study
☒	☐ Functional and/or effective connectivity
☒	☐ Graph analysis
☒	☐ Multivariate modeling or predictive analysis
